# Solitary pulmonary nodule malignancy predictive models applicable to routine clinical practice: a systematic review

**DOI:** 10.1186/s13643-021-01856-6

**Published:** 2021-12-06

**Authors:** Marina Senent-Valero, Julián Librero, María Pastor-Valero

**Affiliations:** 1 Department of Public Health, History of Science and Gynaecology, Faculty of Medicine, Miguel Hernández University, Sant Joan d’Alacant, Alicante, Spain; 2 Navarrabiomed, Complejo Hospitalario de Navarra, UPNA, Pamplona, Spain; 3 Red de Investigación en Servicios de Salud en Enfermedades Crónicas (REDISSEC), Valencia, Spain; 4 CIBER in Epidemiology and Public Health (CIBERESP), Madrid, Spain

**Keywords:** Solitary pulmonary nodule, Prediction models, Lung neoplasms, Clinical setting, Systematic review

## Abstract

**Background:**

Solitary pulmonary nodule (SPN) is a common finding in routine clinical practice when performing chest imaging tests. The vast majority of these nodules are benign, and only a small proportion are malignant. The application of predictive models of nodule malignancy in routine clinical practice would help to achieve better diagnostic management of SPN. The present systematic review was carried out with the purpose of critically assessing studies aimed at developing predictive models of solitary pulmonary nodule (SPN) malignancy from SPN incidentally detected in routine clinical practice.

**Methods:**

We performed a search of available scientific literature until October 2020 in Pubmed, SCOPUS and Cochrane Central databases. The inclusion criteria were observational studies carried out in low-risk population from 35 years old onwards aimed at constructing predictive models of malignancy of pulmonary solitary nodule detected incidentally in routine clinical practice. Studies had to be published in peer-reviewed journals, either in Spanish, Portuguese or English. Exclusion criteria were non-human studies, or predictive models based in high-risk populations, or models based on computational approaches. Exclusion criteria were non-human studies, or predictive models based in high-risk populations, or models based on computational approaches (such as radiomics). We used The Transparent Reporting of a multivariable Prediction model for Individual Prognosis Or Diagnosis (TRIPOD) statement, to describe the type of predictive model included in each study, and The Prediction model Risk Of Bias ASsessment Tool (PROBAST) to evaluate the quality of the selected articles.

**Results:**

A total of 186 references were retrieved, and after applying the exclusion/inclusion criteria, 15 articles remained for the final review. All studies analysed clinical and radiological variables. The most frequent independent predictors of SPN malignancy were, in order of frequency, age, diameter, spiculated edge, calcification and smoking history. Variables such as race, SPN growth rate, emphysema, fibrosis, apical scarring and exposure to asbestos, uranium and radon were not analysed by the majority of the studies. All studies were classified as high risk of bias due to inadequate study designs, selection bias, insufficient population follow-up and lack of external validation, compromising their applicability for clinical practice.

**Conclusions:**

The studies included have been shown to have methodological weaknesses compromising the clinical applicability of the evaluated SPN malignancy predictive models and their potential influence on clinical decision-making for the SPN diagnostic management.

**Systematic review registration:**

PROSPERO CRD42020161559

**Supplementary Information:**

The online version contains supplementary material available at 10.1186/s13643-021-01856-6.

## Background

Solitary pulmonary nodule (SPN), defined as pulmonary opacity up to 30 mm in diameter, is a common finding in routine clinical practice when performing chest imaging tests such as radiographs or computed tomography for any reason [1, 2]. The vast majority of these nodules are benign, and only a small proportion (around 10–20%) are malignant [3, 4]. In a recent cohort study in Spain, after 5 years of follow-up, a prevalence of malignancy of SPN, incidentally detected by chest radiography or computed tomography (CT), of 12.1% and 18.2% respectively, was observed [5]. With the inclusion of CT as a diagnostic test in routine clinical practice, the incidental finding of SPN has increased significantly, leading to the generation of new clinical practice guidelines for its diagnostic management [1, 6–8]. The Fleischner guidelines [6, 7] are based on an exhaustive review of the literature and expert opinion on the diagnostic management of SPN incidentally found on lung CT in patients ≥ 35 years, excluding the high-risk population (screening), immunocompromised or current cancer (of any type) patients.

This guide [7] uses the probability of pre-test malignancy based on individual characteristics of the nodule and the patient, to determine the duration of radiological follow-up. Although nodule size and morphology (higher risk of spiculate edge versus regular edge), remain the dominant factors to predict the risk of SPN malignancy, the Fleischner guidelines consider other additional risk factors such as consistency of the nodule (higher risk in subsolid nodules compared to solid), nodule growth rate, nodule location, higher risk in upper lobes, smokers of ≥ 30 pack-years, exposure to carcinogens (asbestos, uranium and radon), emphysema and/or pulmonary fibrosis, and/or apical scar, family history of lung cancer, over 40 years of age, race (individuals of African descent and Hawaiians being more at risk) and sex (with a higher risk in women with subsolid nodules). Its main recommendations are follow-up with CT at 3 months, positron emission tomography-computed tomography (PET-CT) or biopsy in solid nodules > 8 mm and high-risk patients, for subsolid nodules of > 6 mm follow-up with CT at 3–6 months for part-solid nodules or 6–12 months follow-up for SPN in ground glass. Routine follow-up is recommended in low-risk patients and SPN < 6 mm.

Adherence to these guidelines is considered very important to decrease both over evaluation (prolonged surveillance, multiple biopsies, unnecessary radiation and surgery, etc.) and under evaluation (diagnostic delay). However, compliance with these in routine clinical practice is far from optimal. Studies in the United States reveal breaches ranging from 39 to 73% [4, 9, 10]. In Spain, a significant overvaluation of 72% of the SPN detected by chest radiography was observed, and 61.5% of those detected by CT [2].

Despite the existence of guidelines for the diagnostic management of SPN, when an SPN appears incidentally in routine clinical practice, clinicians tend to adopt a proactive attitude. The key question here is knowing the cancer risk of the SPN detected in the course of routine clinical care—not in a screening setting. For this, it is essential to know and determine the thresholds, conditioned by the characteristics of the patient and the nodule, as a basis to support the decision to continue with additional diagnostic procedures or maintain active surveillance.

Over the years, multivariate predictive models have been designed that are mathematical equations that combine and relate multiple predictors of a particular individual to obtain a pre-test risk/probability of the future presence or occurrence of a particular result [11]. Most predictive models of malignancy of an SPN arise from high-risk populations, with very strict inclusion criteria that therefore render them difficult to extrapolate to usual clinical populations. An exception is the model by McWilliams et al. [12], the “Brock model”, that despite being built from a high-risk population (screening), has been externally validated in a routine clinical practice population and has been shown to be equally valid [13].

Facing clinical intuition/experience as a guide for decision-making in the management of SPN, the application of predictive models of malignancy in routine clinical practice would help to achieve better diagnostic management of SPN. For this, it is necessary to know and evaluate the current state of knowledge in relation to predictive models of malignancy of SPN in the general or low-risk population. In the absence of systematic reviews, the present review was carried out with the purpose of critically analysing studies that have constructed predictive models of malignancy of SPNs found incidentally in routine clinic settings to be applicable in standard clinic contexts.

## Material and methods

### Design

A systematic review of studies for the construction of predictive models of malignancy of SPN applicable in routine clinic settings. The study protocol was registered with the University of York Centre for Reviews and Dissemination International prospective register of systematic reviews (PROSPERO Record CRD42020161559, http://www.crd.york.ac.uk/PROSPERO/).

### Source of data collection

We performed a search for scientific articles from the first available date in the following databases until October 2020: PubMed, SCOPUS and Cochrane Central. The final search equation was developed for use in MEDLINE, adapting it to the rest of the databases consulted, leaving the following: (“solitary pulmonary nodule” [MeSH Terms] OR (“solitary” [All Fields] AND “pulmonary” [All Fields ] AND “nodule” [All Fields]) OR “solitary pulmonary nodule” [All Fields]) AND (prediction [All Fields] AND model [All Fields]) AND ((“lung” [MeSH Terms] OR “lung” [All Fields] OR “pulmonary” [All Fields]) AND (“neoplasms” [MeSH Terms] OR “neoplasms” [All Fields] OR “malignancy” [All Fields])). We also completed the search with an assessment of the bibliographic list of the articles selected, including in the analysis studies that had been identified, but had not been detected in the digital.

### Selection of articles

Inclusion criteria were observational studies carried out in the general population, who are at least 35 years old, in a hospital setting complying with the study objective: construction of predictive models of malignancy of pulmonary solitary nodule detected incidentally in routine clinical practice, studies published in peer-reviewed journals, in Spanish, Portuguese or English. Exclusion criteria: non-human studies, screening for lung cancer, metastatic nodules, models based on computational approaches (such as radiomics) and non-empirical analysis tools were excluded. The selection of articles was carried out independently by 2 authors (MSV and MPV). We prioritized sensitivity over specificity in the selection of the articles. Possible discordance was resolved by consulting a third author (JL) and subsequently consensus among all authors was reached. The inter-observer variability was calculated using Cohen’s kappa coefficient (*K*). These two reviewers carried out an initial screening independently based on the title and abstract of the eligible publications. Duplicates identified through the electronic bibliographic databases were removed. Finally, full articles were retrieved.

### Data extraction

The studies in this review were described considering the following data: first reference author and year of publication, where the study and follow-up were carried out, type of study, characteristics of the population, number of participants, prevalence of malignancy, prevalence of former smokers or active smokers, statistical analysis and predictor variables (Table 1).

**Table 1:** Main characteristics of the studies included in the review

Authors of the models (year of publication)	Location and follow-up of the study	Type of study	Study population	Number of subjects	Prevalence of malignancy (%)	Prevalence of current or past smokers (%)	Statistical methods	Predictor variables
M. Jacob et al. (2020) [26]	Portugal, NR	Retrospective cohort study	Patients who underwent percutaneous CT-guided transthoracic biopsy. Only cases where the biopsy target was less than 3 cm diameters in initial CT evaluation were included. Patients with a clinical record of interstitial lung disease were excluded.	121	53%	Benign nodules: 48% Malignant nodules: 51.9%	Logistic regression analysis	Clinical/radiological characteristics
Chen W et al. (2020) [22]	China, NR	Retrospective case–control study	Patients who had undergone PN resection. The inclusion criterion was patients with PN of ≤ 10 mm in size on preoperative chest high-resolution CT. The exclusion criteria were 10% increase in maximum diameter within 3 months; personal or family history of cancer; or lesions completely calcified.	216	74%	NR	Logistic regression analysis	Clinical/radiological characteristics
Wu Z et al. (2020) [23]	China, NR	Retrospective cohort study	Patients with the radiographic diagnosis of SPNs. All diagnoses of SPNs were pathologically confirmed through operation or biopsy. Patients with multiple nodules or history of lung cancer or extrapulmonary carcinoma were excluded.	721	NR	Benign nodules: 19% Malignant nodules: 28%	Logistic regression analysis	Clinical/radiological characteristics
Chen et al. (2019) [17]	China, NR	Retrospective cohort study	Patients with SPNs who underwent surgical resection to confirm the benignity /malignancy of the nodule. The exclusion criteria were patients with a history of cancer (≤ 5 years ago), immunocompromised patients, PN with a feeding artery and vein typical, PN with intranodular fat or calcium content or absence of the available thin-slice (1 mm) images.	493	43.4%	Benign nodules: 34.05% Malignant Nodules: 33.18%	Logistic regression analysis	Clinical/radiological characteristics and serum biomarkers
Wang et al. (2018) [28]	China,2 years	Retrospective cohort study	Patients who were confirmed with SPNs and had undergone PET/CT. Malignant nodules were confirmed by histopathologic examination of the tissue obtained by surgery or biopsy and benign nodules were confirmed by pathologic diagnosis or clinical follow-up. Patients with the longest diameter of SPNs < 7 mm, a history of primary lung cancer, or related thoracic surgery with distant metastasis were excluded.	177	67.23%	NR	Logistic regression analysis	Clinical/radiological characteristics
She et al. (2017) [24]	China, NR	Retrospective cohort study	Patients with benign/malignant SPNs diagnosed by thin-section CT radiologically confirmed by surgery or biopsy. Subsolid nodules and any indeterminate nodule were excluded.	899	67.3%	19.7%	Logistic regression analysis	Clinical/ radiological characteristics and serum biomarkers
Yang et al. (2017) [29]	China, NR	Retrospective cohort study	Patients with SPNs who underwent CT-guided needle biopsy in their hospital. All of them had biopsy pathology results of benign /malignant nodule. Patients with a history of primary lung cancer were excluded.	1078	66.88%	Benign nodules: 32.4% Malignant nodules: 40% Indeterminate nodules: 37.7%	Logistic regression analysis	Clinical and radiological characteristics
Van Gómez López et al. (2015) [16]	Spain, NR	Retrospective cohort study	Patients with a SPN who underwent a combined whole-body FDG PET/CT imaging and surgical resection of the SPN. A definitive pathologic diagnosis of the SPN, classifying the lesions as benign or malignant, was established.	55	72.7%	NR	Logistic regression analysis	Clinical/ radiological characteristics
Zheng et al. (2015) [18]	China, NR	Retrospective cohort study	Patients with newly discovered SPN found on conventional chest CT scans. A definite benign/malignant diagnosis was obtained based on pathology examination. Patients who were diagnosed as having any cancer within the previous 5 years were excluded, as were those with a history of primary lung cancer or with multiple distant metastases.	846	NR	Benign nodules: 30.05% Malignant nodules: 22.3%	Logistic regression analysis	Clinical/radiological characteristics and serum biomarkers
Zhang et al. (2015) [25]	China, NR	Retrospective cohort study	Patients who underwent surgery /lung resection for histopathological diagnosis of SPN. An exclusion criterion was incomplete data.	294	59.9%	Benign nodules: 31.4% Malignant nodules: 48.3%	Logistic regression analysis	Clinical/radiological characteristics and serum biomarkers
Dong et al. (2013) [15]	China, NR	Retrospective cohort study	Patients with SPNs diagnosed by chest CT scans or X-ray with a histological diagnosis report as a benign or malignant nodule. Exclusion criteria were patients had antineoplastic therapy, radiotherapy or chemotherapy prior to surgery, cancer diagnosis within one year prior to the operation for SPNs; patients had incomplete clinical data; postoperative histological diagnosis of patients was the metastatic cancer of extrapulmonary organs.	1679	77.45%	47%	Logistic regression analysis	Clinical/ radiological characteristics and serum biomarkers
Li et al. (2012) [19]	China, NR	Retrospective cohort study	Patients who had a solitary pulmonary nodule resection to obtain a pathological diagnosis of benignity or malignancy. Patients were excluded if they had a history of pulmonary or extrapulmonary malignancy in 5 years or incomplete data.	371	61.7%	Benign nodules: 32.4% Malignant nodules: 48%	Logistic regression analysis	Clinical and radiological characteristics
Yonemori et al. (2007) [20]	Japan, NR	Retrospective cohort study	Patients who underwent surgery for histopathological diagnosis of SPN. Any SPN diagnosed as metastatic extrapulmonary cancer or any cancer within the past 5 years was excluded.	452	75%	Benign nodules: 47% Malignant nodules: 54%	Logistic regression analysis	Clinical/radiological characteristics and serum biomarkers
Gould et al. (2007) [27]	USA, 2 years	Retrospective cohort study	Patients from 10 geographically diverse VA sites with newly discovered PN seen on chest radiograph. Exclusion criteria included age < 21 years, presence of pregnancy or lactation, weight > 350 to 400 lbs, intercurrent pulmonary infection, thoracic surgery within 6 months, radiotherapy to the chest within 1 year, and life expectancy of < 1 year.	375	54%	Benign nodules: 91% Malignant nodules: 97%	Logistic regression analysis	Clinical and radiological characteristics
Swensen et al. (1997) [21]	USA, 2 years	Retrospective cohort study	They also excluded participants who did not have a qualifying CT scan and/or did not have a definitive diagnosis of an SPN. as malignant or benign established. Patients with newly discovered SPNs detected by chest radiograph or CT scans. Patients who were diagnosed as having any cancer within the past 5 years were excluded. No patients with clinical signs of persistent or recurrent malignant neoplasm or with a history of primary lung cancer were included.	629	23%	Benign nodules: 61% Malignant nodules: 86% Indeterminate nodules: 71%	Logistic regression analysis	Clinical and radiological characteristics

Abbreviations: *SPN*, solitary pulmonary nodule; *PN*, pulmonary nodule; *VA*, Veterans Affairs; *cm*, centimetres; *PET*, positron emission tomography; *FDG PET/CT*, F-fluorodeoxyglucose-positron emission tomography/computed tomography; *NR*, not reported

In Table 2, we present the clinical and radiological variables of the 15 predictive models evaluated. These were described according to the recommendations of the Fleischner guidelines 2017 [7]. The clinical characteristics included: sex, race, emphysema, fibrosis, apical scarring, multiplicity and perifissural nodules; the radiological characteristics included: nodule size, growth rate, morphology, consistency and location. For SPN growth rate, the volume doubling time (1 VDT is equivalent to a 26% increase in diameter) is recommended, being in the 100–400-day range for the majority of solid cancers and on the order of 3–5 years for subsolid cancerous nodules.

**Table 2:** Clinical and radiological variables included in the models described according to Fleischner Guidelines 2017

Models (year)	Clinical characteristics						Radiological characteristics				Variables not included in Fleischner guidelines	Independent predictors of malignancy of SPN in the prediction models
	AGE (mean age)	Sex/race	Smoking history (pack/years)	Family history of lung cancer/history of cancer (%)	Exposure to asbestos, uranium, radon or second-hand smoke	Emphysema/fibrosis/apical scarring/perifissural nodules/multiplicity	Nodule size (diameter mm)	Growth rate^**a**^	Morphology/consistency	Location		
M. Jacob et al. (2020) [26]	64.7 ± 12.3 years (≥ 70 years) Benign nodules: 35.4% Malignant nodules: 64.6%	Male or Female/NR	NR	NR/current extra-pulmonary cancer history: 13.9%	NR	NR/Multiple nodules: Benign nodules: 59.1% Malignant nodules: 40.9%	≤ 30 mm	NR	Margins (Smooth, Lobulated, Spiculated) Calcification Cavitation Sphericity/Solid and subsolid nodules	Central localization	Pleural contact, Air bronchogram, Pulmonary cancer history, Previous TB, Accidental finding, smoking Status.	Age Category, Gender, smoking Status, current Extra pulmonary cancer history, Air bronchogram, nodule size.
Chen W et al. (2020) [22]	51.2 ± 10.9 years Benign nodules: 52.9 ± 10.6 years Malignant nodules: 50.6 ± 11.0 years	Male or Female/NR	Exposure to smoking > 6 months, including second-hand smoke Benign nodules: 16.1% Malignant nodules: 5.6%	NR	(See “smoking history”)	NR/ Incisure surrounding nodules: Benign nodules: 16.1% Malignant nodules: 2.5% Multiple nodules: Benign nodules: 76.8% Malignant nodules: 87.5%	≤ 10 mm	NR	Spiculation, Cavitation sign, Calcification/Solid and subsolid nodules	Left Right Upper Middle Lower	Vascular penetration sign, Pleural adhesions, Long axis, Short axis, Ratio of short-axis to long-axis of the nodule, Nodule density (HU),	Lung nodule density (in HU), vascular penetration sign, nodule type, Incisure surrounding nodules
Wu Z et al. (2020) [23]	Benign nodules: 51.9 ± 11.8 years Malignant nodules: 59.6 ± 9.5 years	Male or Female/NR	(yes/no) Benign nodules: 33% (yes) Malignant nodules: 51%(yes)	NR	NR	NR	< 30 mm	NR	Smooth margin Calcification Nodule density (Patch, Glass, Dense, Cavity, Soft tissue) Clear border /NR	Upper lobe Other lobes	During of smoking Drinking, Superficial lymphadenopathy, Pleural effusion, Profession, others (RCDW, WBC, PLT, Percentage of lymphocyte, monocyte, and basophil, Albumin, globulin, Fibrinogen)	Age, gender, smoking history, drinking history, smooth margin, calcification, clear border
Chen et al. (2019) [17]	Benign nodules: 50.35 ± 10.65 years Malignant nodules: 54.92 ± 9.59 years (range 25-75 years)	Male or Female/NR	(yes/no) Benign nodules: 34.05% (yes) Malignant nodules: 33.18% (yes)	Benign nodules: 13.98% Malignant nodules: 16.82%/History of extrathoracic malignant neoplasm (>5 years ago): 4.32%	NR	Emphysema/NR	8–20 mm	NR	Marginal Spiculation/Solid nodules	·Upper lobe	Pleural Indentation, BMI, Chronic interstitial or obstructive lung disease, significant enhancement, biomarkers (CEA, CA125, CA199, CA724, NSE, SCC, Ferritin)	Age, marginal spiculation, significant enhancement, and pleural indentation
Wang et al. (2018) [28]	Benign nodules: 56.19 ± 10.82 years Malignant nodules: 64.22 ± 9.36 years	Male or Female/NR	(yes/no) Benign nodules: 11% (yes) Malignant nodules: 36% (yes)	NR/ History of cancer: benign nodules: 2%, malignant nodules: 11%.	NR	NR	6–30 mm	NR	Border Lobulation Vascular convergence Pleural retraction Cavity Spiculation Calcification Vacuole Calcification, Spiculation, / Solid and subsolid nodules	RUL RML RLL LUL LLL	Time since quitting, Family cancer history, CT value, SUVmax	Age, lobulation, vascular convergence, pleural retraction, SUVmax
Yang et al. (2017) [29]	55.41 ± 11.94 years (Range, 17–87 years) Benign nodules: 49.01 ± 11.88 years Malignant nodules: 58.22 ± 10.83 years	Male or Female/NR	Benign nodules: 41.03 ± 36.58 pack/years Malignant nodules: 36.09 ± 63.51 pack/years Indeterminate nodules: 32.39 ± 20.50 pack/years	NR/Previous medical history of malignancy lung disease: 5%	NR	NR	4–30 mm	NR	Thin cavitation Thickened cavitation Lobulation Lobulation + spiculation Others: Spiculated protuberances Irregular edge Smooth edge Density Necrosis/ Solid and subsolid nodules	Upper lobe Middle lobe Lower lobe	Previous medical history, extrathoracic disease/ lung Disease excluding malignancy	Gender, age, smoking history, previous extrathoracic disease, previous chronic lung disease except cancer, malignancy history, diameter, lobulation, spiculation, lobulation and spiculation, irregular edges, calcification
She et al. (2017) [24]	58.87 ± 10.74 years	Male or Female/NR	NR	NR/History of cancer: 5.2%	NR	Peripheral emphysema/NR	5–30 mm	NR	Calcification, Spiculation, Cavitation/Solid nodules	·Left lung ·Upper lobe	Family history of cancer, biomarkers (CEA, SCCA) Pleural indentation	Diameter, cancer history, age, spiculation, pleural indentation, calcification, CEA
Van Gómez López et al. (2015) [16]	Benign nodules: 58.0±9.1 years Malignant nodules: 64.2±11.1 years	Male or Female/NR	(yes/no) Benign nodules: 27.3% (yes) Malignant nodules: 63.6% (yes)	NR	NR	NR	< 30 mm	NR	NR/NR	NR	–	SUVmax, age
Zhang et al. (2015) [25]	55.1±10.7 years	Male or Female/	Benign nodules: 162.0±47.8	NR/Previous cancer	NR	NR	≤ 30 mm	NR	Calcification Spiculation Cavitation	LUL LLL RUL	Family history of cancer, biomarkers	Age, smoking history, diameter, spiculation, clear
Yang et al. (2017) [29]	55.41 ± 11.94 years (Range, 17–87 years) Benign nodules: 49.01 ± 11.88 years Malignant nodules: 58.22 ± 10.83 years	Male or Female/NR	Benign nodules: 41.03 ± 36.58 pack/years Malignant nodules: 36.09 ± 63.51 pack/years Indeterminate nodules: 32.39 ± 20.50 pack/years	NR/Previous medical history of malignancy lung disease: 5%	NR	NR	4–30 mm	NR	Thin cavitation Thickened cavitation Lobulation Lobulation + spiculation Others: Spiculated protuberances Irregular edge Smooth edge Density Necrosis/ Solid and subsolid nodules	Upper lobe Middle lobe Lower lobe	Previous medical history, extrathoracic disease/ lung Disease excluding malignancy	Gender, age, smoking history, previous extrathoracic disease, previous chronic lung disease except cancer, malignancy history, diameter, lobulation, spiculation, lobulation and spiculation, irregular edges, calcification
She et al. (2017) [24]	58.87 ± 10.74 years	Male or Female/NR	NR	NR/History of cancer: 5.2%	NR	Peripheral emphysema/NR	5–30 mm	NR	Calcification, Spiculation, Cavitation/Solid nodules	·Left lung ·Upper lobe	Family history of cancer, biomarkers (CEA, SCCA) Pleural indentation	Diameter, cancer history, age, spiculation, pleural indentation, calcification, CEA
Van Gómez López et al. (2015) [16]	Benign nodules: 58.0±9.1 years Malignant nodules: 64.2±11.1 years	Male or Female/NR	(yes/no) Benign nodules: 27.3% (yes) Malignant nodules: 63.6% (yes)	NR	NR	NR	< 30 mm	NR	NR/NR	NR	-	SUVmax, age
Zhang et al. (2015) [25]	55.1±10.7 years (Range, 32-80 years) Benign nodules: 50.11±10.15 years Malignant nodules: 61.01±11.36 years	Male or Female/NR	Benign nodules: 162.0±47.8 pieces-year Malignant nodules: 258.9±71.1pieces-year	NR/Previous cancer history: 1.7%	NR	NR	≤ 30 mm	NR	Calcification Spiculation Cavitation Lobulation Others: Pleural retraction sign Clear border Vascular convergence sign/NR	LUL LLL RUL RML RLL	Family history of cancer, biomarkers (CEA, NSE CYFRA 21-1)	Age, smoking history, diameter, spiculation, clear border, CYFRA 21-1
Zheng et al. (2015) [18]	Benign nodules: 52.5 ± 12.0 years Malignant nodules: 58.7 ± 11.4 years	Male or Female/NR	Pieces-year ≥ 400 %: Benign nodules: 24.45% Malignant nodules: 19.45%	NR	NR	NR	< 30 mm	NR	Calcification Spiculation Cavitation Lobulation Others: satellite lesions/Subsolid nodules	RUL RML RLL LUL LLL	Haemoglobin, total protein, albumin, ALP, creatinine, LDH, calcium, biomarkers (CEA), family tumour history, BMI, past related diseases, symptoms, FEV_1,_ Pleural tail, central pixel attenuation, enhancement attenuation value, enlarged lymph nodes, pleural effusion	Model with Nodules < 50% GGO: age, symptoms, serum total protein, diameter, lobulation, calcification. Model with Nodules ≥ 50% GGO: sex, FEV_1_ %, diameter, calcification
Dong et al. (2013) [15]	58.12 years (Range, 32–80 years) Benign nodules: 50.11±10.15 years Malignant nodules: 61.01±11.36 years	Male or Female/NR	468.15 Pieces-year pieces-year Malignant nodules: 258.9±71.1pieces-year	NR/Previous history of history: 1.7%	NR	NR	< 30 mm	NR	Calcification Spiculation Cavitation Lobulation Others: Pleural retraction sign Clear border Vascular convergence sign/NR	LUL LLL RUL RML RLL	Family cancer history, biomarkers (CEA, NSE CYFRA 21-1)	Age, smoking history, CEA, CYFRA 21-1, border, CYFRA 21-1
Zheng et al. (2015) [18]	Benign nodules: 52.5 ± 12.0 years Malignant nodules: 58.7 ± 11.4 years	Male or Female/NR	Pieces-year ≥400 %: Benign nodules: 24.45% Malignant nodules: 19.45%	NR	NR	NR	< 30 mm	NR	Calcification Spiculation Cavitation Lobulation Others: satellite lesions/Subsolid nodules	RUL RML RLL LUL LLL	Haemoglobin, total protein, albumin, ALP, creatinine, LDH, calcium, biomarkers (CEA), family tumour history, BMI, past related diseases, symptoms, FEV_1,_ Pleural tail, central pixel attenuation, enhancement attenuation value, enlarged lymph nodes, pleural effusion	Model with Nodules < 50% GGO: age, symptoms, serum total protein, diameter, lobulation, calcification Model with Nodules ≥ 50% GGO: sex, FEV_1_ %, diameter, calcification
Dong et al. (2013) [15]	58.12 years	Male or Female/NR	468.15 Pieces-year	NR/Previous history of	NR	NR	< 30 mm	NR	Calcification Spiculation Cavitation	LUL LLL RUL	Family cancer history, biomarkers	Age, smoking history, CEA, CYFRA 21-1,
				Malignant tumour > 1 year ago:3.45%					Lobulation Others: Clear border Satellite lesions Pleura retraction sign /NR	RML RLL	(NSE, CEA, CYFRA 21-1, CA125, SCC), histological diagnosis malignant	family history of cancer, diameter, lobulation, calcification, spiculation, clear border, satellite lesions
Li et al. (2012) [19]	57.1 years Benign nodules: 48.0 ± 14.6 years Malignant nodules: 61.2 ± 13.1 years	Male or Female/NR	Benign nodules: 169.8 ± 328.3 pieces-year Malignant nodules: 260.6 ± 410.3 pieces-year	NR/Previous cancer history > 5 years ago: 2.25%	NR	NR	< 30 mm	NR	Calcification Spiculation Cavitation Lobulation Others: Pleural retraction sign Clear border Vascular convergence/NR	Upper lobe Non-upper lobe Left Right	Family history of cancer	Age, diameter, spiculation, family cancer history, calcification, clear border
Yonemori et al. (2007) [20]	62 years Benign nodules: 58 years Malignant nodules: 64 years	Male or Female/ NR	Benign nodules: 19 pack-years Malignant nodules: 24 pack-years	NR/Other cancer > 5 years ago: 2.21%	NR	NR	< 30 mm	NR	Calcification Spiculation Cavitation Lobulation Others: CT bronchus sign/NR	LUL Lingular segment LLL RUL RML RLL Right	WCC, serum CRP, biomarkers (CEA)	Calcification, spiculation, Bronchus sign, CEA, CRP
Gould et al. (2007) [27]	65.9 ± 10.7 years Benign nodules: 62 years Malignant nodules: 68 years	Male or Female/NR	Benign nodules: 46 pack-years Malignant nodules: 63 pack-years	NR/History of other cancers: 9.06%	NR	NR	7–30 mm	NR	“definitely malignant”^b^/NR	Upper lobe /right lung	Number of years since quitting smoking, time since diagnosis of lung/other cancer	Years since quitting smoking, smoking history, age, diameter
Swensen et al. (1997) [21]	Benign nodules: 60 years (range, 15-82 years) Malignant nodules: 65 years (range, 35-87 years)	Male or Female/NR	Benign nodules: 24 pack-years Malignant nodules: 45 pack-years Indeterminate nodules: 32 pack-years	Of extrathoracic malignancy: 2.86%	6 subjects with SPN had been exposed to asbestos	NR	4–30 mm	NR	Calcification, Spiculation, Cavitation, Lobulation Others: Entirely smooth, Spiculated or shaggy/NR	·Central/peripheral ·Upper lobe ·Location lobe: RUL RML RLL LUL LLL Lingula	Pleural tail, Air bronchogram, residence > 192 km from clinic	Age, history of smoking, Remote history (>5 years) of extrathoracic cancer, diameter, spiculation, upper lobe

Notes: ^a^100-400 days for volume doubling time (solid nodules); 3–5 years (subsolid nodules). ^b^“Definitely malignant” is described in Gould et al. article as: ‘readers of chest radiographs were asked to provide a radiographic diagnosis on a 5-point Likert scale that ranged between “definitely benign” and “definitely malignant.” We therefore used a radiographic diagnosis of “definitely malignant” as a proxy for spiculation

Abbreviations :*LLL*, left lower lobe; *RUL*, right upper lobe; *RML*, right middle lobe; *RLL*, right lower lobe; *LUL*, left upper lobe; *GGO*, ground-glass opacity; *BMI*, body mass index; *LDH*, lactate dehydrogenase; *ALP*, alkaline phosphatase; *NSE*, neuron-specific enolase; *CEA*, carcinoembryonic antigen; *CYFRA 21-1*, cytokeratin 19-fragment marker; *SCC*, squamous cell carcinoma; *CA125*, carbohydrate antigen 125; *CA199*, carbohydrate antigen 199; *CA724*, carbohydrate antigen 724; *FEV*_*1*_, forced expiratory volume 1; *WCC*, leukocytes; *CRP*, *RCDW*, red cell distribution width; *WBC*, white blood cell; *PLT*, platelet counts; *SUVmax*, the maximum of standardized uptake value; *CRP*, C-reactive protein; *HU*, Hounsfield units; *TB*, tuberculosis; *NR*, not reported

Additional files 1 and 2 show the external validations; those carried out by the authors themselves and by other authors, respectively. In turn, Additional file 1 describes the results of applying models developed by other authors to the same sample. Furthermore, in both Appendices, we use the Transparent Reporting of a multivariable Prediction model for Individual Prognosis Or Diagnosis (TRIPOD) statement [14] to describe the type of predictive model included in each study included in our review, as well as the results of the discrimination and the calibration of these.

Finally, in Additional file 3 we describe the predictive mathematical models of each study evaluated.

### Quality of research

The Prediction model Risk Of Bias ASsessment Tool (PROBAST) was used to assess the quality of the selected articles [11], with the aim of providing a structured judgement of the risk of bias, thereby allowing the analysis of the applicability and transferability of predictive models to clinical practice. It contains 20 items on potential biases distributed in 4 domains/dimensions (participants, predictors, results and analysis). Applicability is analysed for participants, predictor and outcome domains. The response templates for each model are reflected in Additional file 4.

**Fig. 1:**
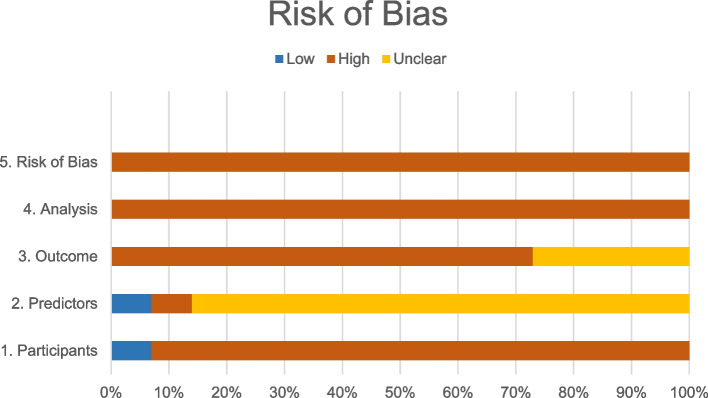
Percentages of the risk of bias of the studies according to PROBAST

**Fig. 2:**
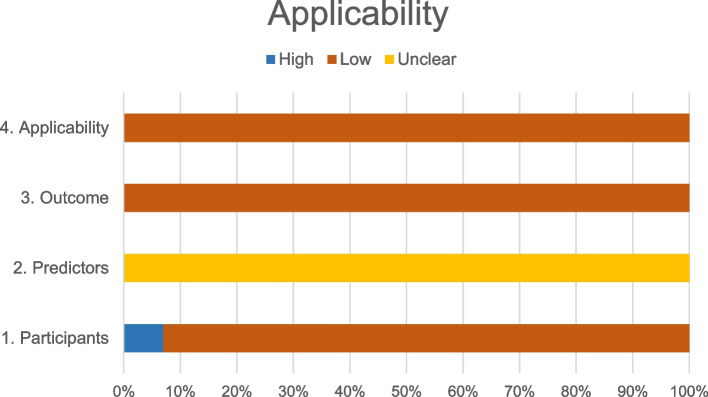
Percentages of the Applicability of the studies according to PROBAST

In Table 3, as in Fig. 1 and Fig. 2, following the table format suggested by PROBAST [11], we have presented the quality results, representing the risk of bias, the applicability and the final global assessment, respectively.

**Table 3:** Quality of the models of the review according to PROBAST tool

Study, year	ROB^**a**^				Applicability^**b**^			Overall^**c**^	
	Participants	Predictors	Outcome	Analysis	Participants	Predictors	Outcome	ROB	Applicability
Swensen et al., 1997 [21]	+	?	−	−	+	?	−	−	−
Gould et al., 2007 [27]	−	−	−	−	−	?	−	−	−
Yonemori et al., 2007 [20]	−	+	−	−	−	?	−	−	−
Li et al., 2012 [19]	−	?	−	−	−	?	−	−	−
Dong et al. 2013 [15]	−	?	−	−	−	?	−	−	−
Zhang et al., 2015 [25]	−	?	?	−	−	?	−	−	−
Zheng et al., 2015 [18]	−	?	?	−	−	?	−	−	−
Van Gómez López et al., 2015 [16]	−	?	−	−	−	?	−	−	−
Yang et al., 2017 [29]	−	?	?	−	−	?	−	−	−
She et al., 2017 [24]	−	?	?	−	−	?	−	−	−
Wang et al., 2018 [28]	−	?	−	−	−	?	−	−	−
Chen et al., 2019 [17]	−	?	−	−	−	?	−	−	−
Wu Z et al. (2020) [23]	−	?	−	.	−	?	−	−	−
Chen W et al. (2020) [22]	−	?	−	−	−	?	−	−	−
M. Jacob et al. (2020) [26]	−	?	−	−	−	?	−	−	−

Notes: “+” indicates low ROB/low concern regarding applicability; “−” indicates high ROB/high concern regarding applicability; and “?” indicates unclear ROB/unclear concern regarding applicability. ^a^ Obtaining each domain of risk of bias is established based on the responses of their respective items (Appendix D) as follows: if all items are answered with "Yes", the domain is at low risk of bias. If in at least one item the answer is “Unclear” and the rest of the items are “Yes”, the risk of bias is unclear. If the answer is “No” in at least one item, independently of the other answers, the domain is at high risk of bias. ^b^ The applicability of each domain is established by consensus among the authors. ^c^ The final overall assessment is expressed as follows: low risk of bias if all domains have low risk of bias; high risk of bias in case at least one domain presents high risk of bias; if the risk is not clear in at least one domain and all the other domains are low risk of bias, the final assessment remains unclear. Ditto for applicability

Abbreviations: *PROBAST*, Prediction model Risk Of Bias Assessment Tool; *ROB*, risk of bias

## Results

**Fig. 3:**
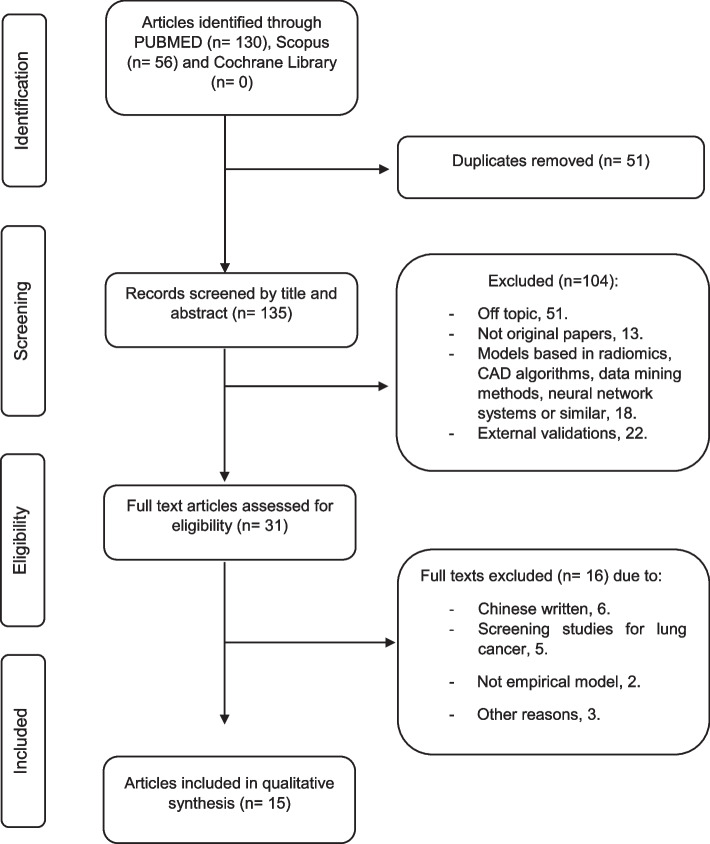
Flow chart outlining study selection

Using the described search criteria, 186 references were identified (56 Scopus, 130 PubMed), of which 51 duplicates were removed. On evaluating the Abstract and title, 104 articles were eliminated and the inter-observer reliability, Cohen’s kappa coefficient, was 0.75 (the authors agreed over the inclusion of 26 articles, excluding 97 and disagreed over 12, of which they eventually accepted 5 and rejected 7 as a result of subsequent consensus among the 3 authors). We retrieved and analysed a final sample of 31 full-text articles. The inter-observer kappa coefficient was 0.87 (the authors agreed over the inclusion of 15 articles and the exclusion of 14, and disagreed over 2, finally rejecting both as a result of subsequent consensus among the 3 authors), leaving 15 articles in the final review (Fig. 3). The quality evaluation of the studies was carried out in pairs in the same way as the selection of the articles with a kappa coefficient > 80%”. The topicality of the articles was calculated using the Burton–Kebler semi-period, which showed that the references had a median age of 5 years, and the Price Index, which showed that 67% of documents were less than 5 years old.

The main characteristics of the studies are shown in Table 1. All were retrospective studies, 2 were carried out in the USA, 11 in China and Japan, 1 in Portugal and1 in Spain. The largest sample size was that of Dong et al. [15], with a cohort of 1679 subjects, and the smallest that of van Gómez López et al. [16] with 55. In all studies, the study population were patients diagnosed with an SPN for the first time in routine clinic settings, in 3 from an imaging test (X-ray or CT/PET-CT of the chest), and in 12 from those sent to surgery/biopsy for histopathological diagnosis. In most studies, the exclusion criteria were previous history of cancer in the last 5 years or fewer, diagnosis of lung cancer or metastasis, and incomplete patient data. In 5 studies [17–21], participants with a previous history of cancer in the past 5 years were excluded, one [15] excluded those in just the previous year, and two excluded patients with a history of cancer but did not specify the time period [22, 23].

All studies analysed clinical and radiological variables, also including biomarkers in 6 studies [15, 17, 18, 20, 24, 25]. The prevalence of malignancy of the nodules ranged from 23 to 77.45% and that of current or past smokers ranged from 19 to 91% in benign nodules, and from 22.3 to 97% in malignant nodules.

Table 2 shows that all the models evaluated included risk factors such as sex, age and the diameter of the SPN. All studies included the morphology and location of the SPN except one [16] and all studies included smoking habits except two [24, 26]. Only one study [21] included exposure to asbestos, none included exposure to radon or uranium, and only one [22] included passive exposure to tobacco smoke. Emphysema was collected in 2 studies [17, 24], and family history of lung cancer was collected in only one [17]. Only one study [22] described perifissural nodules, and two studies [22, 26] included multiple nodules. Race, growth rate of the nodule, fibrosis and apical scarring were not reported in any of the studies.

In relation to nodule consistency, the majority of the studies (*n* = 8) did not specify the nodule consistency [15, 16, 19–21, 23, 25, 27] whereas the other 7 studies included this information: 1 study reported subsolid nodules [22], 2 studies collected patients with only solid nodules [17, 24], and 4 [22, 26, 28, 29] included both (solid and subsolid nodules). However, only 1 [22] of the 7 studies analysed the predictive risk of nodule consistency and found that mixed ground-glass nodules showed a higher risk of malignancy compared to solid nodules.

All studies in the review included individuals of both sexes (1:1), except two [16, 27] in which the male sex predominated and one [22] in which the female sex predominated. The mean age of patients with benign nodules ranged from 48 to 62 years, and that of patients with malignant nodules ranged from 50 to 68 years. Previous cancer history was present in 11 articles [15, 17, 19–21, 24–29] and ranged from 1.7 to 13.9%.

Finally, the independent predictors of malignancy of SPN that were identified most frequently in the models were age (*n* = 13), SPN diameter (*n* = 9), edge spiculation (*n* = 8), nodule calcification (*n* = 7) and smoking history (*n* = 6). Also found, among others, were defined edge of the nodule (*n* = 4), lobulation of the nodule (*n* = 4), previous history of cancer (*n* = 4) and the carcinoembryonic antigen (CEA) biomarker (*n* = 3).

In Additional file 1, according to the TRIPOD classification [14], four studies were type 1a [16, 22, 26, 28], one study was type 1b [27], 5 were type 2a [15, 18, 21, 23, 24] and 5 were type 3 [17, 19, 20, 25, 29]. The sample size of the validations ranged from 120 to 344 participants. The discrimination of the models ranged from 0.599 [27] to 0.910 [25]. Only 1 of the 15 models was calibrated [17], with a calibration of 0.928.

In Additional file 2, according to the TRIPOD classification [14], the 17 studies were type 4. Validations were carried out in the USA [30–34], Asia (China, Japan, Korea) [35–40], one in the UK [41], one in Brazil [42], one in the Netherlands [43] and another in Italy [44], and in 2, it was not specified [45, 46]. The prevalence of malignancy ranged from 25 to 85.6%, with one study not providing this information, and the prevalence of current or past smokers ranged from 11.8 to 89%, although this was not reported in 4 studies [39, 40, 42, 46]. The size of the validations ranged from 86 to 702 participants. The area under the curve (AUC) of all the models ranged from 0.53 to 0.89. Only 5 [31, 33, 34, 37, 43] of the 17 studies estimated the calibration of the models, 2 of these models [19, 21] underestimated the probability of malignancy; while another model [27] underestimated in 2 studies [31, 37] and overestimated in one [34].

It should be noted that only 3 [19, 21, 27] of the 15 studies included were validated by other authors, with Swensen et al. [21] being the most validated model.

### Assessment of methodological quality

The models risk of bias was assessed using the PROBAST tool [11] (Table 3, Figures 1 and 2). Regarding the Participants dimension, only 3 of the 15 studies were rated as appropriate by PROBAST, as case–control studies nested in a cohort [21, 27, 28]. The rest were non-nested case–control studies [15–17, 19, 20, 22, 24–26]; in 3 [18, 23, 29], the type of study was not clear. Only two studies [21, 28] included all patients in routine clinical practice; the other studies selected those who underwent surgery/biopsy or who had suspected malignancy. Regarding the Predictors dimension, the possibility in any study of the result of malignancy being known prior to evaluation as recommended by PROBAST could not be ruled out; only one [20] specified that the results were unknown. Furthermore, in one study [27] predictors were not evaluated similarly because radiographs were studied by different radiologists. Regarding the Results dimension, the method of determining malignancy (surgery, biopsy or follow-up) was not adequate in 8 studies [15–17, 19, 20, 22, 23, 26] and was ambiguously estimated in 5 [18, 24, 25, 27, 29], only being correct in two [21, 28]. None reported whether the measurement of the results was performed without the predictors analysed being known. Furthermore, the time interval between the evaluation of the variables and obtaining the result was not adequate in 3 studies [21, 27, 28], in the rest it is unknown. Regarding the Analysis dimension, only 8 presented an adequate number of participants providing relevant results [20, 29] or an adequate number of events per variable [15, 16, 22, 24, 27, 28], as 3 could not be specified due to lack of data [18, 21, 23] or inadequate data in 3 [17, 19, 25]. In 5 studies [17, 18, 23, 26, 29], continuous variables were categorized/ dichotomized, and in only one [17] were data imputation techniques used for missing values.

Discrimination (AUC) and calibration (using the calibration slope ± Hosmer–Lemeshow test) were evaluated in 5 studies [17, 21, 23, 24, 27] , while in 4 others [15, 20, 22, 25], only the calibration (Hosmer–Lemeshow test) was evaluated; and in the rest [16, 18, 19, 26, 28, 29], only discrimination was evaluated. Only in 2 [17, 24] were bootstrapping techniques used to avoid overestimation of the model. In 8 of the 15 models [15–17, 19, 21, 23, 27], the predictor weights of the models were assigned according to the results obtained from the multivariate analysis; of the rest, in 3 it was not clear due to lack of information [18, 28, 29] and it was not correct in 4 others due to errors in the mathematical equation [18] and because the assigned weights did not coincide with the multivariate analysis [22, 24, 26] (see Additional file 3).

All studies were classified with a high risk of bias compromising their applicability (Fig. 2).

## Discussion

Our systematic review describes and evaluates published predictive models of solitary pulmonary nodule (SPN) malignancy built from SPN incidentally encountered in routine clinical practice. The findings of this study showed that, there is an increasing scientific interest in developing new predictive models; 67% of the article publication date was less than 5 years old; however, the design of the predictive models assessed showed important methodological deficiencies which compromises their clinical applicability. To describe the models, we followed The Fleischner Society recommendations [7] for the management of incidentally found solitary pulmonary nodules (solid or subsolid). To evaluate the applicability and transferability of the predictive models to clinical practice we used the PROBAST tool [11].

To our knowledge, this is the first systematic review of studies that develop predictive models of SPN malignancy in routine clinical practice, with 73% of them (11/15) performed in Asian populations. A recent prospective study of a multiethnic cohort corroborated that Native Hawaiians and African Americans have twice the excess risk of developing lung cancer, with a low number of cigarettes consumed, compared to Japanese Americans and Latinos [47]; however, in this review, we did not find studies on predictive models based on Hawaiians or African Americans. Moreover, the Fleischner guidelines [7] consider race to be a risk factor for SPN malignancy; but this risk factor was not included in any of the models reviewed.

Age, followed by the size of the nodule (diameter) were the most frequently identified independent predictors in 13 studies and 9 respectively. This is in line with the scientific evidence [6, 7] showing that, with increased age and SPN diameter, the risk of malignancy also increases.

Fleischner recommendations [7] on nodule size are to use the average diameter as the average of long- and short-axis diameters, both of which should be obtained on the same transverse, coronal or sagittal reconstructed image, which more accurately reflects three-dimensional tumour volume. Of the 15 models, only 4 described how the nodule diameter was measured. Thus, 3 studies [16, 22, 28] only reported that the images of the nodule were acquired in 3-D dimensional mode, and 1 [22] that the long and short axes of the nodules were measured, and the ratio of the short to long axis was calculated. Nodule diameter was not identified as an independent predictor risk factor of SPN malignancy in any of these studies.

As regards **sex**, differences have been observed in the clinical management of SPNs, with diagnostic delays identified, leading to a therapeutic delay, and greater radiation in women [48]. In our review, all studies included a female population, and in one [18], the predictive model with the highest proportion of ground glass (≥ 50%) identified being a woman as an independent predictor.

As regards **morphology**, SPN **spiculation** appears as a frequent predictor in almost all studies [15, 17, 19–21, 24, 25, 29], with lobulation also being significant, as a final predictor of SPN malignancy in 4 studies [15, 18, 28, 29].

Regarding **calcification**, central/lamellar/diffuse/popcorn calcifications suggest benignity, while dotted patterns/eccentric localization suggest malignancy. Calcification was predictive in 7 models [15, 18–20, 23, 24, 29]. However, as the calcification pattern was not taken into account, nodules with calcification indicating benign characteristics were treated in the same manner as if the pattern suggested malignancy, possibly creating bias in terms of the prediction of malignancy.

Although **smoking** is considered the highest risk criterion, it was only identified as a predictor in 6 of the models [15, 21, 23, 25, 27, 29]. In the rest [16–20, 22, 24, 26, 28], it was perhaps not identified because the proportion of smokers/ex-smokers was low and the malignant SPNs showed a greater proportion of adenocarcinomas, a histological pattern that is less related to this exposure.

The previous history of any type of cancer in family members was collected in 6 studies [15, 18, 19, 24, 25, 28] and was identified as a malignancy predictor in 2 [15, 19]. Furthermore, the previous personal history of cancer was collected in 11 studies [15, 17, 19–21, 24–29], and in 4 of the models [21, 24, 26, 29], it was found to be a predictive factor of malignancy. Despite genetic susceptibility has been described previously, concluding that there is an association between a previous history of cancer in first-degree relatives, and increased risk of lung cancer in both sexes [49], only one study [17] evaluated the previous history of lung cancer in relatives and found that it was not a predictor of malignancy.

Some models found that CEA [15, 20, 24] and CYFRA 21-1 [15, 25] biomarkers were final predictors of malignancy; however, none of the studies performed external validations, nor do the Fleischner guidelines include them as risk factors for malignancy. Further studies are required to assess their future importance in routine clinical practice

Exposure to other carcinogens (asbestos, uranium, radon) has been described as a risk factor for lung cancer [7, 50]. However, only one study collected exposure to asbestos [21] but did not identify it as a predictor. Passive exposure to tobacco is one of the causes of lung cancer and it has been shown that 40% of children, 33% of non-smoking men and 35% of non-smoking women are exposed worldwide [51], only one study [22] analysed it and it was not found that passive exposure to tobacco smoke was an independent predictor of malignancy.

According to Fleischner guidelines, lung cancers occur more frequently in the upper lobes. However, although all studies collected the nodule location, only one study conducted in the USA [21] identified it as an independent predictor. In China, there is a high prevalence of tuberculosis and other granulomatous diseases, typically located in the upper lobes. Most of the studies in this review involved the Asian population, without a relationship between nodule location and malignancy being observed.

Finally, emphysema, considered a risk factor [7], was identified in 2 articles [17, 24], although neither was predictive. Chronic obstructive pulmonary disease (COPD) was evaluated in a single study [17] but was not identified as a predictor. In another study [29], a final predictor was the history of chronic lung disease, but the type of disease was not specified. A recent meta-analysis confirms that this comorbidity is frequent in patients with lung cancer and that both this and emphysema increase the level of risk, especially in smokers with heavy tobacco use [52].

### Assessment of the prediction model risk of bias

We followed the PROBAST guidelines on potential biases distributed in 4 domains (participants, predictors, results and analysis) to set out several methodological deficiencies of the studies included [11].

There is clear disagreement between the prevalence of SPN malignancy found in the models included in this review (between 23 and 77.45%) and the prevalence in daily clinical practice (between 12.1 and 18.2%) [5]. This is probably due to the fact that most models are based on the population referred for surgery/biopsy, with consequent selection bias, since there is an important group of the population attended to in routine clinic settings—those considered to be at lower risk of malignancy and less likely to be sent to surgery/biopsy—not included in most of the models studied. This selection bias occurs in all the studies except three [21, 27, 28], which used a case–control design nested in a cohort study, also including those that only required radiological follow-up. The rest describe themselves as retrospective cohort studies [15–17, 19, 20, 22, 24–26], and in three, the type of study is not well established [18, 23, 29].

According to PROBAST [11], the prospective cohort study is considered the optimal design [11] with low risk of bias, since it allows all the information on the potential predictors (exposures) to be collected before the potential outcome, thus reducing selection or interviewer biases. Non-nested case–control studies in a cohort select a population from a study designed for another purpose, and therefore have a higher risk of bias. In line with the results obtained by Collins et al. [53], the models are seldom prospective and usually use information from populations intended for a completely different purpose.

Nodule consistency (solid, subsolid) is a determining factor when predicting SPN malignancy. The stability of solid nodules is estimated over a period of 2 years [6, 7], whereas in subsolids, it is 5 years [7]. Thus, longer initial follow-up intervals and longer total follow-up periods are recommended for subsolid nodules than for solid nodules. Bearing this in mind, this was insufficient in the 3 studies that followed up [21, 27, 28] with 2 years of follow-up, respectively. The remaining studies [15, 16, 18–25, 27, 29] did not specify whether they followed up.

In some models, there was categorization of continuous variables: in one [17], the values of the biomarkers were dichotomized; in others, it was the smoking history (≥ 30 pack-years) [29], (≥ 400 pieces-year) [18]; and in one, it was the age (≥ 70 years) [26]. This establishes an arbitrary cut-off point, from which a different risk level is established, causing loss of information, so that predictive capacity is lost [11].

In most of the studies, the analysis does not mention patients with missing data. These are interpreted as having been omitted, meaning that the analysis performed is an “available/complete case analysis”. This is the most frequent type of analysis in predictive models and is the one which we suppose was in 14 of the 15 studies in which this information was not reported. The exclusion of missing data leads to biases in the association of the predictors with the result and skews the performance of the model because after the exclusion of cases with incomplete information, the selected subpopulation may not be representative of the population. Only one study [17] took into account the missing data, and used the multiple imputation technique as recommended by PROBAST, with a lower risk of bias, and is considered the best method described [11].

The optimal sample size for binary prediction models is considered to be a minimum of 100 events (preferably ≥ 200) for external validations [53], with 10–15 events per variable (EPV) (better ≥ 20) [54] for development models [11]. This was the case in only 8 studies [15, 16, 20, 22, 24, 27–29].

The external validation of any development model in an independent sample is essential to demonstrate its satisfactory performance, i.e. applicability and transferability in clinical practice. One of the most important limitations of the models created so far is the lack of external validations. External authors have only validated 3 models [19, 21, 27] (Additional file 2), the most frequently evaluated being that of Swensen et al. [21], which has presented good discrimination in all of them, with values greater than 0.75 [55]. Although there are studies that have created models and have externally validated them with very promising results [17, 20, 25], there are no studies as yet that corroborate the results obtained.

In some studies [15, 20, 22, 25], the Hosmer–Lemeshow Test was the only calibration method used. However, it is not without limitations: large sample sizes can generate erroneous results and it does not reveal the magnitude of the difference between the predicted values and the observed values [55]. This does not happen with the calibration slope (the method most recommended by PROBAST), which was performed in only 5 articles [17, 21, 23, 24, 27].

The 15 models analysed showed low clinical applicability due to the high probability of bias. In normal practice, models that do not present selection biases are required, ones that reflect all possible malignancy risk profiles (from none to all) that may occur in a patient with an SPN found incidentally. Some models are not explicit in the exclusion of patients with a recent history of cancer (the last 5 years) [16, 24, 25, 27, 29]; possibly, they are more likely to experience a tumour recurrence/metastasis, thus overestimating the predictive values. In other cases, only solid nodules are included [17, 24] and cannot be applied to patients with subsolid nodules, and vice versa. Other recommendations on predictors and their measurements are that they should be standard and applicable to the clinical setting; specifically, biomarkers may not always be available.

### Limitations and strengths of this review

The heterogeneity of the studies did not allow for a meta-analysis. Only studies in English, Spanish or Portuguese were included. These languages allow a wide coverage of more than 90% of articles in the literature, however, we discarded 6 articles written in other languages that could have been relevant.

Additionally, when we used the search equation, there were a large number of articles that were not ultimately relevant to the study objective. This may have been due to the lack of specific descriptors (MeSH), which meant that we had to use Pubmed to search for Title and Abstract fields.

The final number was limited (*n* = 15) and most involved high-risk populations, which limits the extrapolation of the results from the models identified to routine clinic practice.

Another limitation is that our literature search was carried out only in three databases (Pubmed, Scopus and Cochrane Central) not including for example important databases such as Embase. However, Scopus is a good alternative having the largest number of health articles which constitutes approximately 90% of the articles processed by PubMed, and more than 97% of the total titles processed by Embase [56]. Therefore, we believe that if there was a risk of publication bias from missing other key databases this was minimal.

The strengths of this review are the rigorous use of standard tools of proven methodological quality to evaluate the proposed models, and the independent selection and review of the articles included their quality, with high concordance among researchers in a relevant area of knowledge, as reflected in the Burton–Kebler Index and the Price Index. We attempted to minimize bias in the review by adhering to a registered protocol and following the PRISMA statement [57].

## Conclusions

Our results indicate that most of the predictive models were built mainly from retrospective studies with poor levels of methodological quality, rendering their applicability in routine clinical practice difficult.

Although there is scientific evidence on multiple factors which determine the risk of malignancy of a SPN, important factors were not considered by most of the studies, such as nodule consistency; growth rate; race; emphysema; fibrosis; exposure to asbestos, uranium and radon; or passive tobacco smoke; among others. Moreover, the evaluation of the studies included in this paper leads us to underline the importance of identifying the risk factors of malignancy of solitary pulmonary nodule in different populations.

Efforts should be channelled towards epidemiological studies with prospective designs, and roust methodology representing the general population that uses clinical services.

We believe that these results highlight key information for clinicians when deciding how to use these models to aid in the diagnostic and therapeutic management of solitary pulmonary nodules.

### Supplementary Information

**Additional file 1.** Types of prediction models included in the revision according to TRIPOD Statement and their validations.

**Additional file 2.** External validation of the included models by different authors from those who created the models.

**Additional file 3.** Mathematical equations of the included models.

**Additional file 4.** Items included in each domain of PROBAST quality.

## Data Availability

Not applicable.

## Abbreviations

SPN: Solitary pulmonary nodule
PROBAST: Prediction model Risk Of Bias ASsessment Tool
TRIPOD: Transparent Reporting of a multivariable Prediction model for Individual Prognosis Or Diagnosis
CT: Computed tomography
PET: Positron emission tomography
CEA: Carcinoembryonic antigen
COPD: Chronic obstructive pulmonary disease
AUC: Area under the curve
EPV: Events per variable
VDT: Volume doubling time
