# Correction: Solitary pulmonary nodule malignancy predictive models applicable to routine clinical practice: a systematic review

**DOI:** 10.1186/s13643-024-02627-9

**Published:** 2024-08-10

**Authors:** Marina Senent‑Valero, Julian Librero, Maria Pastor‑Valero

**Affiliations:** 1 Department of Public Health, History of Science and Gynaecology, Faculty of Medicine, Miguel Hernandez University, Alicante, Sant Joan d’Alacant Spain; 2 Navarrabiomed, Complejo Hospitalario de Navarra, UPNA, Pamplona, Spain; 3 Red de Investigacion en Servicios de Salud en Enfermedades Cronicas (REDISSEC), Valencia, Spain; 4 CIBER in Epidemiology and Public Health (CIBERESP), Madrid, Spain

**Correction: Syst Rev 10, 308 (2021)**

10.1186/s13643-021-01856-6

Following publication of the original article [1], the authors identified an error in the funding section. Below is the correct funding.

Incorrect funding statement:

This research did not receive any specific grant from funding agencies in the public, commercial, or not-for-profit sectors.

Correct funding statement:

This study was supported by the Fondo de Investigaciones Sanitarias, Instituto de Salud Carlos III (Plan Nacional de I + D + I) PI18/00258 and co-funded by the Fondo Europeo de Desarrollo Regional (FEDER).

The original article [1] has been corrected.
