# Supplementary material for: Solitary pulmonary nodule malignancy predictive models applicable to routine clinical practice: a systematic review
Source: Syst Rev. 2021 Dec 6;10:308. doi: 10.1186/s13643-021-01856-6 (PMC8650360; doi:10.1186/s13643-021-01856-6)
Supplement: Supplementary file 1 — Additional file 1. Types of prediction models included in the revision according to TRIPOD Statement and their validations. [file 13643_2021_1856_MOESM1_ESM.docx]

**Appendix A** Types of prediction models included in the revision according to TRIPOD Statement and their validations

| **Authors of the model**  **(year)** | **Types of prediction models by TRIPOD statement^1^** | **Validation sample size** | **AUC^2^** | **Calibration^3^** |
| --- | --- | --- | --- | --- |
| M. Jacob et al.  (2020) (26) | 1a |  |  |  |
| Chen W et al.  (2020) (23) | 1a |  |  |  |
| Wu Z et al.  (2020) (22) | 2a | - | - | - |
| Chen et al.  019) (21) | 3 | (without CEA level) :216 | Chen et al.: 0.847 (C-index) | 0.928 |
|  |  | (with CEA level) :216 | Chen et al.: 0.848 (C-index) | 0.866 |
| Wang et al.  (2018) (29) | 1a | - | - | - |
| She et al.  (2017) (24) | 2a | - | - | - |
| Yang et al.  (2017) (28) | 3 | 344 | Yang et al.: 0.784 | - |
|  |  |  | Swensen et al.:0.649 |  |
|  |  |  | Gould et al.: 0.599 |  |
| Van Gómez López et al. (2015) (16) | 1a | - | - | - |
| Zheng et al.  (2015) (20) | 2a | - | - | - |
| Zhang et al.  (2015) (25) | 3 | 120 | Zhang et al.: 0.910 | - |
|  |  |  | Zhang et al. (without CYFRA 21-1): 0.812 |  |
|  |  |  | Swensen et al.: 0.752 |  |
|  |  |  | Gould et al.: 0.730 |  |
|  |  |  | Li et al.: 0.833 |  |
| Li et al.  (2012) (19) | 3 | 145 | Li et al.: 0.874 | - |
|  |  |  | Swensen et al.:0.784 |  |
|  |  |  | Gould et al.: 0.754 |  |

(Continued)

**Appendix A** (*Continued*)

| **Authors of the model**  **(year)** | **Types of prediction models by TRIPOD statement^1^** | **Validation sample size** | **AUC^2^** | **Calibration^3^** |
| --- | --- | --- | --- | --- |
| Dong et al.  (2013) (15) | 2a | - | - | - |
| Yonemori et al.  (2007) (18) | 3 | 148 | Yonemori et al.:0.840 | - |
| Gould et al.  (2007) (27) | 1b | - | - | - |
| Swensen et al.  (1997) (17) | 2a | - | - | - |

**Abbreviations**: CEA, carcinoembryonic antigen; CYFRA 21-1, Cytokeratin 19-fragment marker; TRIPOD, Transparent Reporting of a multivariable Prediction model for Individual Prognosis Or Diagnosis; AUC, the Area Under the Curve.

**Notes**: ^1^Types of prediction models by TRIPOD statement: **Type 1** corresponds to the construction of a predictive model without performing internal / external validation techniques (in **type 1a** the performance of the model is evaluated in the same group in which the model is developed, and therefore, it is an apparent performance ; in **type 1b**, resampling (for example bootstrapping or cross-validation techniques) is used to evaluate the performance and optimism of the model). **Type 2** corresponds to the construction of predictive models and performance an internal validation. In this type of model, the sample of participants is divided into two groups: one to develop the model and the other to validate it; if this division of the participants is carried out randomly, we are dealing with a **type 2a** study and if it is carried out in a non-randomized way (for example, by location or time), it is a **type 2b** study. According to TRIPOD, type 2b can also be considered as a development model halfway between internal validation and external validation. **Type 3** corresponds to the construction of a predictive model and the performance of an external validation in a separate population (that is, the model is developed in a group of participants and validated in a separate group other than the one used to create the model). ^2^AUC = **Discrimination** is the ability of the model to assign, on pairs of randomly selected subjects, one from the group with malignancy and the other without it, the correct result (greater probability in the group with malignancy). For binary results, the area under the ROC curve (AUC), or C statistic, is the most frequently used discrimination measure. ^3^ **Calibration** is a measure that expresses the agreement between the observed results and the model predictions. The most common calibration measurements are the calibration slope and the Hosmer-Lemeshow test.
