# Supplementary material for: Solitary pulmonary nodule malignancy predictive models applicable to routine clinical practice: a systematic review
Source: Syst Rev. 2021 Dec 6;10:308. doi: 10.1186/s13643-021-01856-6 (PMC8650360; doi:10.1186/s13643-021-01856-6)
Supplement: Supplementary file 2 — Additional file 2. External validation of the included models by different authors from those who created the models. [file 13643_2021_1856_MOESM2_ESM.docx]

**Appendix B** External validation of the included models by different authors from those who created the models

| **Authors**  **(year)** | **Types of prediction models by TRIPOD statement^1^** | **Study population** | **Prevalence of malignancy** | **Prevalence of**  **active or former smokers** | **Validation sample size** | **Prediction model** | **AUC^2^** | **Calibration^3^** |
| --- | --- | --- | --- | --- | --- | --- | --- | --- |
| Li et al.  (2020) (40) | 4 | Chinese patients with SPN who had surgery and had a clear pathological diagnosis. | 85.6% | NR | 496 | Swensen et al. | 0.62 | - |
|  |  |  |  |  |  | Gould et al. | 0.62 |  |
|  |  |  |  |  |  | Li et al. | 0.63 |  |
| Cui et al.  (2019) (39) | 4 | Chinese patients with newly detected SPN on CT scans that had been confirmed based on histopathological result or remained stable for at least 2 years. | 72.4% | NR | 277 | Swensen et al. | 0.77 | **-** |
|  |  |  |  |  |  | Gould et al. | 0.66 |  |
| Yang B et al.  (2018) (38) | 4 | Patients with biopsy-proven pulmonary lung nodules at a single centre in Korea. | 77.2% | 39% | 242 | Swensen et al. | 0.61 | - |
|  |  |  |  |  |  | Gould et al. | 0.60 |  |
| Hammer M et al.  (2017) (30) | 4 | USA patients with large nodules at high risk for lung cancer (non-screening cohort). | 69% | 84% | 86 | Swensen et al. | 0.62 | - |
|  |  |  |  |  |  | Gould et al. | 0.59 |  |
|  |  |  |  |  |  | Li et al. | 0.53 |  |
| Soardi G et al.  (2016) (44) | 4 | Italian patients with SPN with a definitive diagnosis by | 54.5% | 52% | 200 | Swensen et al. | 0.60 | - |

(Continued)

**Appendix B** (*Continued*)

| **Authors**  **(year)** | **Types of prediction models by TRIPOD statement1^1^** | **Study population** | **Prevalence of malignancy** | **Prevalence of**  **active or former smokers** | **Validation sample size** | **Prediction model** | **AUC^2^** | **Calibration^3^** |
| --- | --- | --- | --- | --- | --- | --- | --- | --- |
|  |  | biopsy or stability/volume reduction. |  |  |  |  |  |  |
| Talwar A et al.  (2016) (31) | 4 | Non-screening USA population with PNs (incidental finding on a chest CT and patients with either a known or prior cancer within the last 5 years scanned either as a staging or follow-up scan or scanned for another reason). | 46% | 65% | 702 | Swensen et al. | 0.58 | This model underestimated the probability of malignancy in 1^st^ 2^nd^ and 3^th^ quintiles. It overestimated the probability of malignancy in 4^th^ and 5^th^ quintiles. |
|  |  |  |  |  |  | Gould et al. | 0.62 | This model underestimated the probability of malignancy in 1^st^ 2^nd^ and 3^th^ quintiles. It overestimated the probability of malignancy in 4^th^ and 5^th^ quintiles. |
| Perandini S et al.  (2015) (46) | 4 | Patients with a newly discovered solid SPN with a definitive diagnosis by biopsy or by means of serial volume assessment found in electronic medical records. | 54.7% | NR | 285 | Swensen et al. | 0.77 | - |
|  |  |  |  |  |  | Li et al. | 0.88 |  |
| Al-Almeri A et al.  (2015) (41) | 4 | UK patients with PN detected in routine clinical practice. | 40.6% | 76.2% | 244 | Swensen et al. | 0.89 | - |
|  |  |  |  |  |  | Gould et al. | 0.73 |  |

(Continued)

**Appendix B** (*Continued*)

| **Authors**  **(year)** | **Types of prediction models by TRIPOD statement1^1^** | **Study population** | **Prevalence of malignancy** | **Prevalence of**  **active or former smokers** | **Validation sample size** | **Prediction model** | **AUC^2^** | **Calibration^3^** |
| --- | --- | --- | --- | --- | --- | --- | --- | --- |
| Tanner et al.  (2015) (32) | 4 | USA patients with a PN with a definitive diagnosis (identified by querying databases). | 25% | 73% | 377 | Swensen et al. | 0.77 | - |
|  |  |  |  |  |  | Gould et al. | 0.74 |  |
| Perandini et al.  (2014) (45) | 4 | Patients with SPN found in CT images from the hospital medical records. | 54.8% | 11.8% | 288 | Swensen et al. | 0.76 | - |
| Zhang X et al.  (2014) (37) | 4 | Patients with SPN who underwent surgical resection, mainly from the south of China. | 81.2% | 31,8% | 154 | Swensen et al. | 0.75 | This model underestimated the probability of malignancy. |
|  |  |  |  |  |  | Gould et al. | 0.72 | This model underestimated the probability of malignancy. |
|  |  |  |  |  |  | Li et al. | 0.80 | This model underestimated the probability of malignancy in all quintiles except the 4^th^ quintile. |
| Shinohara S et al.  (2014) (36) | 4 | Japanese patients with SPN who underwent surgical resection. | 84.2% | 61.8% | 241 | Swensen et al. | 0.67 | - |
| Xiao F et al.  (2013) (35) | 4 | Patients with SPN confirmed by CT who underwent surgical procedure in China-Japan. | 72.8% | 37.4% | 107 | Swensen et al. | 0.78 | - |
|  |  |  |  |  |  | Gould et al. | 0.68 |  |
|  |  |  |  |  |  | Li et al. | 0.81 |  |
| Melo CB et al.  (2012) (42) | 4 | Brazilian patients submitted to resection of SPN in Brazil. | NR | NR | 110 | Swensen et al. | 0.79 | - |
|  |  |  |  |  |  | Gould et al. | 0.69 |  |

(Continued)

**Appendix B** (*Continued*)

| **Authors**  **(year)** | **Types of prediction models by TRIPOD statement^1^** | **Study population** | **Prevalence of malignancy** | **Prevalence of**  **active or former smokers** | **Validation sample size** | **Prediction model** | **AUC^2^** | **Calibration^3^** |
| --- | --- | --- | --- | --- | --- | --- | --- | --- |
| Isbell et al.  (2011) (33) | 4 | USA patients who underwent resection of a PN. | 73% | 74% | 189 | Swensen et al. | 0.79 | This model underestimated the probability of malignancy in 4^th^ and 5^th^ quintiles, the other quintiles were well calibrated. |
| Schultz E et al.  (2008) (34) | 4 | USA patients with SPN discovered incidentally in chest CT, who underwent surgery. | 45% | 89% | 151 | Swensen et al. | 0.80 | This model underestimated the probability of malignancy in all quintiles except the 1^st.^. |
|  |  |  |  |  |  | Gould et al. | 0.73 | This model overestimated the probability of malignancy. |
| Herder et al. (2005) (43) | 4 | Dutch patients with an indeterminate SPN, which had been detected during normal clinical work ,who had been referred for FDG-PET-CT. | 57.5% | 74.5% | 106 | Swensen et al. | 0.79 | This model tended to underestimate the probability of malignancy, particularly at lower probabilities. |

**Abbreviations**: SPN, Solitary Pulmonary Nodule; PN, Pulmonary Nodule; UK, United Kingdom; USA, United States of America; CT, Computed Tomography; FDG-PET, F-fluorodeoxyglucose-Positron Emission Tomography/Computed Tomography; NR, Not Reported; TRIPOD, Transparent Reporting of a multivariable Prediction model for Individual Prognosis Or Diagnosis ; AUC, the Area Under the Curve.

**Notes**: ^1^Types of prediction models by TRIPOD statement: **Type 4** corresponds to an external validation, in a separate population, of a previously constructed model (a new model is not created, only an external validation of an existing model is performed). ^2^AUC = **Discrimination** is the ability of the model to assign, on pairs of randomly selected subjects, one from the group with malignancy and the other without it, the correct result (greater probability in the group with malignancy). For binary results, the area under the ROC curve (AUC), or C statistic, is the most frequently used discrimination measure. ^3^ **Calibration** is a measure that expresses the agreement between the observed results and the model predictions. The most common calibration measurements are the calibration slope and the Hosmer-Lemeshow test.
