# Supplementary material for: Solitary pulmonary nodule malignancy predictive models applicable to routine clinical practice: a systematic review
Source: Syst Rev. 2021 Dec 6;10:308. doi: 10.1186/s13643-021-01856-6 (PMC8650360; doi:10.1186/s13643-021-01856-6)
Supplement: Supplementary file 3 — Additional file 3. Mathematical equations of the included models. [file 13643_2021_1856_MOESM3_ESM.docx]

**Appendix C** Mathematical equations of the included models

**M. JACOB ET AL. MODEL**

- Pre-test probability of a malignant SPN = e^x^/(1+e^x^)
- x = −8.61 + (1.56 × **Age Category**) + (1.87 × **Gender**) + (1.26 × **Smoking Status**) + (1.28 × **Current ExtraPulmonary Cancer**) + (1.27 × **Air Bronchogram**) + (0.22 × **Nodule Size**).

where e is the base of natural logarithms, while x is the regression coefﬁcient in the logistic regression; The rest is not reported.

In this study, the assigned weight of one predictor in the ﬁnal model do not correspond to the result from the reported multivariable analysis: the weight assigned to “Current ExtraPulmonary Cancer” variable is 1.28, to obtain this value the corresponding OR would be 3.59 (on the other hand, in the multivariate analysis table the OR = 8.94), therefore the assigned weight should be **2.19**.

**CHEN W ET AL. MODEL**

- Pre-test probability of a malignant SPN = e^x^/(1+e^x^)
- x = -2.957 - (0.004 x **lung nodule density**) + (1.096 x **vascular penetration sign**) + (1.198 x **nodule type**) - (1.811 x **incisure**).

where e is the base of natural logarithms, while x is the regression coefﬁcient in the logistic regression; lung nodule density indicates the lung nodule density in Hounsfield Units; vascular penetration sign (1 = if vascular penetration sign is present, 0 = otherwise), nodule type (1 = mixed Ground Glass Nodule (GGN), solid or pure GGN= 0); incisure (1 = if incisure surrounding nodules are present, otherwise = 0).

In this study, the assigned weights of the predictors in the ﬁnal model do not correspond to the results from the reported multivariable analysis: the weight assigned to “Nodule density” variable is 0.004, to obtain this value the corresponding OR would be 1.004, (on the other hand, in the multivariate analysis table the OR = 0.995), therefore the assigned weight should be **-0.005**; the weight assigned to “vascular penetration sign” variable is 1.096, to obtain this value the corresponding OR would be 2.99, (on the other hand, in the multivariate analysis table the OR = 3.49), therefore the assigned weight should be **1.25**; the weight assigned to “Nodule Type” variable is 1.198, to obtain this value the corresponding OR would be 2.99, (on the other hand, in the multivariate analysis table the OR = 4.27), therefore the assigned weight should be **1.451**; the weight assigned to “incisure” variable is 1.811, to obtain this value the corresponding OR would be 6.11, (on the other hand, in the multivariate analysis table the OR = 0.179), therefore the assigned weight should be **-1.720**.

**WU Z ET AL. MODEL**

The **Wu Z et al. model** is defined by the equations:

- Pre-test probability of a malignant SPN = e^x^/(1+e^x^)
- x = − 2.8107 + (1.2454 × **smoking history**) + (− 1.4055 × **edge**) + (0.077 1 × **age**) + (− 1.0 728 × **gender**) + (− 0 .6228 × **clear border**) + (− 1.3319  ×  **calcification**) + (0.6 890  ×  **drinking** **history**).

where e is the base of natural logarithms, while x is the regression coefﬁcient in the logistic regression; age indicates the patient’s age in years; gender (1 = if the patient is male, 0 = otherwise); smoking history (1 = current or former smoker, otherwise = 0); calcification (1 = if calciﬁcation is present in the SPN, otherwise = 0); drinking history (1= if the patient has history of drinking, 0 = otherwise); edge (1= if smooth edge is present in the SPN, otherwise = 0); clear border (1= if a clear border is present in the SPN, otherwise = 0).

**CHEN ET AL. MODEL**

The **Chen et al. model** is defined by the equations:

- Pre-test probability of a malignant SPN = e^x^/(1+e^x^)
- x = − 4.439 + (0.034 × **age**) + (1.347 × **marginal spiculation**) + (2.210 × **signiﬁcant enhancement**) + (1.211× **pleural indentation**).

where e is the base of the natural logarithms, while x is the regression coefﬁcient in the logistic regression; age indicates the patient’s age in years; marginal spiculation (1 = if spiculated appearance is present in the SPN, otherwise = 0); signiﬁcant enhancement (1= if significant enhancement is present, otherwise = 0); pleural indentation (1 = if pleural indentation is present, otherwise = 0).

They described an alternative model adding CEA level:

- Pre-test probability of a malignant SPN = e^x^/(1+e^x^)
- x = − 4.688 + (0.036× **age**) + (1.396 × **marginal spiculation**) + (2.174 × **signiﬁcant enhancement**) + (1.235 × **pleural indentation**) + (1.572 × **CEA**).

where e is the base of the natural logarithms, while x is the regression coefﬁcient in the logistic regression; age indicates the patient’s age in years; marginal spiculation (1 = if spiculated appearance is present in the SPN, otherwise = 0); signiﬁcant enhancement (1= if significant enhancement is present, otherwise = 0); pleural indentation (1 = if pleural indentation is present, otherwise = 0); CEA = serum CEA level (ng/mL).

This model is also expressed as a normogram as follows:


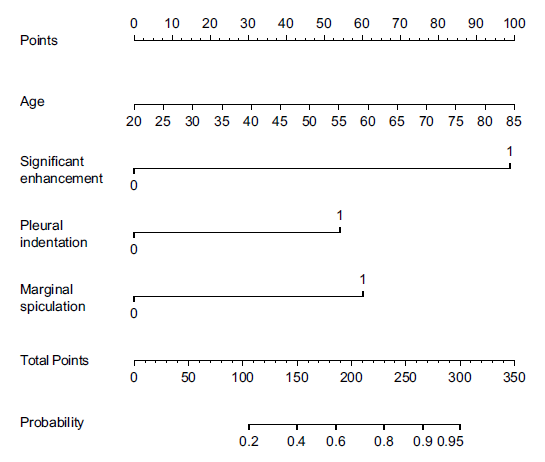


To obtain the nomogram-predicted probability, locate the patient values at each axis, and draw a vertical line to the “Points” axis to determine the number of points attributed to each variable value, determine total number of points for all variables, and locate the sum on the “Total Points” line to assess the individual probability of lung cancer.

**WANG ET AL. MODEL**

- Pre-test probability of a malignant SPN = e^x^/(1+e^x^)
- 𝑥 = − 7.363 + (0.079 × **age**) + (1.90 × **lobulation**) + (1.024 × **vascular convergence**) + (1.530 × **pleural retraction**) + (0.359 × **SUVmax**),

where 𝑒 is the base of the natural logarithms; while x is the regression coefﬁcient in the logistic regression; age indicates the patient’s age in years; lobulation (1 = if lobulation is present in the SPN, otherwise = 0); vascular convergence (1 = if vascular convergence is present in the SPN, otherwise = 0); pleural retraction (1 = if pleural retraction is present in the SPN, otherwise = 0); SUVmax is the maximum uptake value on the PET.

**SHE ET AL. MODEL**

The **She et al. model** is defined by the equations:

- Pre-test probability of a malignant SPN = e^x^/(1+e^x^)
- x = - 5.4175 + (0.8149 × **Log_10_ CEA**) + (1.0447 × **diameter**) + (2.5978 × **cancer history**) + (0.0518 × **age**) + (1.7166 × **spiculation**) + (0.3986 × **pleural indentation**) - (2.2549 × **calcification**).

where e is the base of the natural logarithms, while x is the regression coefﬁcient in the logistic regression; Log_10_CEA = the log base 10 transformations of serum CEA value (ng/mL); the diameter of the SPN is measured in millimetres (mm); cancer history (1= if cancer history is present, otherwise = 0); age indicates the patient’s age in years; spiculation (1 = if spiculated appearance is present in the SPN, otherwise = 0); pleural indentation (1 = if pleural indentation is present, otherwise = 0); calcification (1 = if calciﬁcation is present in the SPN, otherwise = 0).

In this study, the assigned weights of three predictors in the ﬁnal model do not correspond to the results from the reported multivariable analysis: the weight assigned to “cancer history” variable is 2.5978, to obtain this value the corresponding OR would be 13.43, (on the other hand, in the multivariate analysis table the OR = 12.82), therefore the assigned weight should be **2.55**; the weight assigned to the “CEA” variable in the model is 0.8149, to obtain this value the corresponding OR would be 2,251 (on the other hand, in the multivariate analysis table the OR = 1.09), therefore, the weight should be **0.086**; the weight assigned to the diameter variable in the model is 1.0447, to obtain this value the corresponding OR would be 2.84 (on the other hand, in the multivariate analysis table the OR = 1.11) therefore, the weight should be **0.095**.


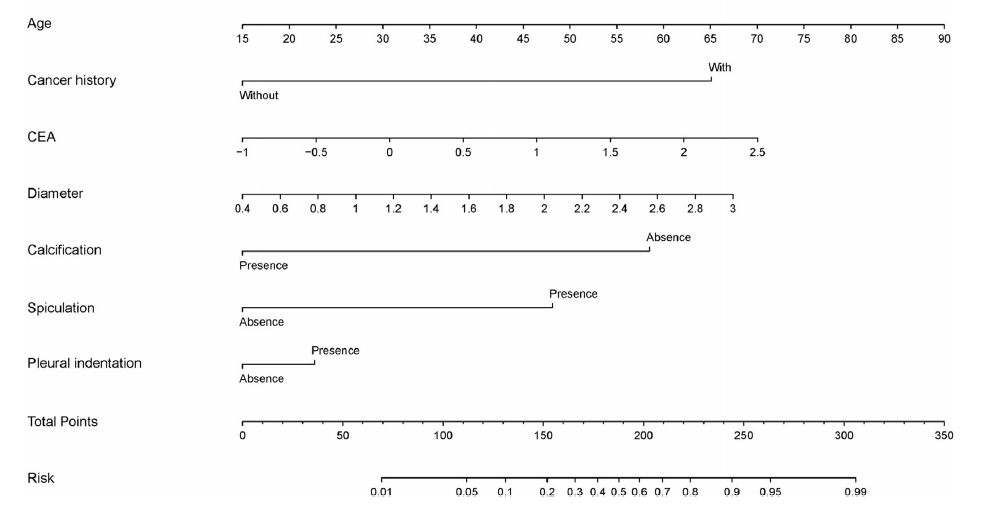
This model is also expressed as a normogram as follows:

To obtain the nomogram-predicted probability, locate the patient values at each axis, and draw a vertical line to the “Points” axis to determine the number of points attributed to each variable value, determine total number of points for all variables, and locate the sum on the “Total Points” line to assess the individual probability of lung cancer, CEA: Log10 (the serum carcinoembryonic antigen value).

**YANG ET AL. MODEL**

The **Yang et al. model** is defined by the equations:

- Pre-test probability of a malignant SPN = e^x^/(1+e^x^)
- x = − 6.173 + (1.207 × **gender**) + (0.580 ×**age**) + (0.520 × **pack-years**) – (0.226 × **previous extrathoracic disease**) – (0.685× **previous chronic lung disease except cancer**) + (2.739 × **malignancy history**) + (0.933 × **diameter**) + (0.702 × **lobulation**) + (0.466 × **spiculation**) + (21.060 × **lobulation and spiculation**) – (1.428 × **irregular edges**) – (2.062 × **calcification**).

where e is the base of the natural logarithms, while x is the regression coefﬁcient in the logistic regression; gender (1 = if the patient is male, 2 = if the patient is female); age indicates the patient’s age in years; pack-years indicates the pack-years of smoking; Previous extrathoracic disease indicates the previous medical history (1 = if it is present, 0 = if it is not present); Previous chronic lung disease except cancer (1= if a previous chronic lung disease except cancer is present, if not = 0); malignancy history ( 1 = if malignancy history is present, otherwise = 0); the diameter of the SPN is measured in millimetres (mm); lobulation (1 = if lobulation is present in the SPN, otherwise = 0); spiculation (1 = if spiculated appearance is present in the SPN, otherwise = 0); lobulation and spiculation ( 1= if both are present in the SPN, otherwise = 0); irregular edges (1= if irregular edges are present in the SPN, otherwise = 0); calcification (1 = if calciﬁcation is present in the SPN, otherwise = 0).

We cannot confirm if the predictors and their assigned weights in the ﬁnal model correspond to the results from the reported multivariable analysis because Yang et al. did not report the univariate and multivariate tables in their study.

**VAN GÓMEZ LÓPEZ ET AL. MODEL**

- Pre-test probability of a malignant SPN = e^x^/(1+e^x^)
- x = − 3.767+ (0.489× **SUVmax**) + (0.052× **age**)

where e is the base of the natural logarithms, while x is the regression coefﬁcient in the logistic regression; age indicates the patient’s age in years; SUVmax is the maximum uptake value on the PET.

**ZHENG ET AL. MODEL**

The **Zheng et al. model** is defined by two equations:

1. The clinical prediction model for malignancy in SPNs with less than 50% ground glass opacity (GGO) is defined by the equations:

- Pre-test probability of a malignant SPN = e^x^/(1+e^x^)
- x = - 7.442 + (0.051 × **age**) + (0.711 × **presence of symptoms**) + (0.066 × **total protein** **concentration**) + (0.032 × **diameter**) + (1.071 × **lobulation**) **-** (1.220 × **calciﬁed nodes**).

where e is the base of the natural logarithms, while x is the regression coefﬁcient in the logistic regression; age indicates the patient’s age in years; presence of symptoms (1= if the patient has related symptoms*, 0 = if the patient has not related symptoms); total protein concentration = serum total protein concentration (g/L); the diameter of the SPN is measured in millimetres (mm); lobulation (1 = if lobulation is present in the SPN, otherwise = 0); calciﬁed nodes (1 = if calciﬁcation is present in the SPN, otherwise = 0).

*these symptoms are described in Zheng et al. article as: “lung cancer-related symptoms, such as cough, shortness of breath, hemoptysis, chest pain, fever, and so forth”.

1. The clinical prediction model for malignancy in SPNs with 50% or greater ground glass opacity (GGO) is defined by the equations:

- Pre-test probability of a malignant SPN = e^x^/(1+e^x^)
- x = - 6.192 **+** (0.924 × **sex**) + (0.042 × **FEV1%)** + (0.131 × **diameter**) **-** (2.424 × **calciﬁed** **nodes**).

where e is the base of the natural logarithms, while x is the regression coefﬁcient in the logistic regression; sex (1 = if the patient is male, 2= if the patient is female); FEV1 = Forced expiratory volume 1 (%); the diameter of the SPN is measured in millimetres (mm); calciﬁed nodes (1 = if calciﬁcation is present in the SPN, otherwise = 0).

In both models, an error in the symbol that precedes two variables has been corrected (marked in bold), in coherence with the Odds ratios reported in the multivariate analysis.

**ZHANG ET AL. MODEL**

The **Zhang et al. model** is defined by the equations:

- Pre-test probability of a malignant SPN = e^x^/(1+e^x^)
- x = −14.417 + (0.111 × **age**) + (1.009 × **smoking history**) + (2.597 × **nodule diameter**) + (1.056 × **spiculation**) + (−1.258 × **clear border**) + (1.184 × **CYFRA 21-1**).

where e is the base of the natural logarithms, while x is the regression coefﬁcient in the logistic regression; age indicates the patient’s age in years; history of smoking (1 = current or former smoker, otherwise = 0) nodule diameter refers to the maximum nodule diameter measured by chest radiography or CT prior to surgery (in cm); spiculation (1 = if spiculated appearance is present in the SPN, otherwise = 0); clear border (1 = if clear border is present in the SPN, otherwise = 0); CYFRA 21-1 = serum CYFRA 21-1 level (ng/mL).

**DONG ET AL. MODEL**

The **Dong et al. model** is defined by the equations:

- Pre-test probability of a malignant SPN = e^x^/(1+e^x^)
- x = − 4.294 + (0.035 × **age**) + (0.221 × **CEA**) + (0.200 × **CYFRA 21-1**) + (1.029 × **smoking**) + (0.974 × **family history of cancer**) + (0.633 × **diameter**) + (−1.631 × **clear border**) + (−1.923 × **satellite lesions**) + (2.673 × **lobulation**) + (−3.295 × **calciﬁcation**) + (2.027 × **spiculation**).

where e is the base of the natural logarithms, while x is the regression coefﬁcient in the logistic regression; age indicates the patient’s age in years; the diameter of the SPN is measured in centimetres (cm); CEA = serum CEA level (ng/mL); CYFRA 21-1 = serum CYFRA 21-1 level (ng/mL); Smoking indicates smoking history (1= if smoking history is present, otherwise = 0); family cancer history (1 = if family cancer history is present, otherwise = 0); clear border (1 = if clear border is present in the SPN, otherwise = 0); satellite lesions (1 = if satellite lesions are present in the SPN, otherwise = 0); lobulation (1 = if lobulation is present in the SPN, otherwise = 0); calciﬁcation (1 = if calciﬁcation is present in the SPN, otherwise = 0); spiculation (1 = if spiculated appearance is present in the SPN, otherwise = 0).

**LI ET AL. MODEL**

The **Li et al. model** is defined by the equations:

- Pre-test probability of a malignant SPN = e^x^/(1+e^x^)
- x = - 4.496 + (0.07 × **age**) + (0.676 × **diameter**) + (0.736 × **spiculation**) + (1.267 × **family history of** **cancer**) - (1.615 × **calcification**) – (1.408 × **border**).

where e is the natural logarithm, while x is the regression coefﬁcient in the logistic regression; age indicates the patient’s age in years; diameter indicates the largest nodule measurement (in cm) reported on initial chest radiograph or CT scan; spiculation (1 = if spiculated appearance is present, otherwise = 0); family cancer history (1 = if family history of cancer exist, otherwise = 0); calciﬁcation (1 = if calcification is present, otherwise = 0); border (1= if clear border is present, otherwise = 0).

**YONEMORI ET AL. MODEL**

The **Yonemori et al. model** is defined by the equations:

- Pre-test probability of a benign SPN = e^x^/(1+e^x^)
- x = 3.7009 + (3.0705 × **calciﬁcation**) + (-1.3243 × **CT bronchus sign**) + (- 5.3399 × **spiculation**) + (-1.16 × **√CEA**) + (-1.4987 × **CRP**).

where e is the base of the natural logarithm, while x is the regression coefﬁcient in the logistic regression; calciﬁcation (1 = if calciﬁcation is present in the SPN, otherwise = 0); CT bronchus sign (1 = if CT bronchus sign is present, otherwise = 0); spiculation (1 = if spiculated appearance is present, otherwise = 0); CEA = serum CEA level (ng/mL); CRP = serum CRP level (mg/L).

They constructed an alternative model in case the biological parameters were not available:

- Pre-test probability of a benign SPN = e^x^/(1+e^x^)
- x = 1.084 + (2.7851 × **calciﬁcation**) + (-1.1795 × **CT bronchus sign**) + (-5.4481 × **spiculation**).

where e is the base of the natural logarithm, while x is the regression coefﬁcient in the logistic regression; calciﬁcation (1 = if calciﬁcation is present in the SPN, otherwise = 0); CT bronchus sign (1 = if CT bronchus sign is present, otherwise = 0); spiculation (1 = if spiculated appearance is present, otherwise = 0).

In this case, the study of Yonemori et al. has created a model which predicts the probability of benignancy instead of malignancy of the SPN. To obtain the probability of malignancy you have to calculate the complementary probability (1- probability of benignancy).

We cannot confirm if the predictors and their assigned weights in the ﬁnal model correspond to the results from the reported multivariable analysis because Yonemori et al. did not report the univariate and multivariate tables in their study.

**GOULD ET AL. MODEL**

The **Gould et al. model (also called VA model)** is defined by the equations:

- Pre-test probability of a malignant SPN = e^x^/(1+e^x^)
- X = - 8.404+ (2.061× **smoke**) + (0.779 × **age10**) + (0.112 × **diameter**) - (0.567 × **yearsquit10**).

where e is the base of the natural logarithm, while x is the regression coefﬁcient in the logistic regression; smoke indicates smoking history (1 = current or former smoker, 0 = never smoker); age10 indicates age in years at the time of nodule identification, divided by 10; diameter indicates the largest nodule measurement (in mm) reported on initial chest radiograph or CT scan; yearsquit10 indicates the number of years since quitting smoking, divided by 10 (0 indicates not applicable).

VA model calculator is available on: <https://magarray.com/calculator-va/>

**SWENSEN ET AL. MODEL**

The **Swensen et al. model (also called The Mayo Clinic model)** is defined by the equations:

- Pre-test probability of a malignant SPN = e^x^/(1+e^x^)
- x = - 6.8272 + (0.0391× **age**) + (0.7917 × **smoke**) +(1.3388 × **cancer**) + (0.1274 × **diameter**) + (1.0407 × **spiculation**) + (0.7838 × **upper lobe**).

where e is the base of the natural logarithm, while x is the regression coefﬁcient in the logistic regression; age indicates the patient’s age in years; smoke indicates smoking history (1 = current or former smoker, 0 = never smoker); cancer indicates history of an extrathoracic cancer >5 years before nodule identification (1 = yes, otherwise = 0); diameter indicates the largest nodule measurement (in mm) reported on initial chest radiograph or CT scan; spiculation (1 = if spiculated appearance is present, otherwise = 0); and upper lobe is location of the nodule within the upper lobe (1 = yes, 0 = no).

Mayo Clinic model calculator is available on: <https://reference.medscape.com/calculator/solitary-pulmonary-nodule-risk> .
