# Supplementary material for: Solitary pulmonary nodule malignancy predictive models applicable to routine clinical practice: a systematic review
Source: Syst Rev. 2021 Dec 6;10:308. doi: 10.1186/s13643-021-01856-6 (PMC8650360; doi:10.1186/s13643-021-01856-6)
Supplement: Supplementary file 4 — Additional file 4. Items included in each domain of PROBAST quality. [file 13643_2021_1856_MOESM4_ESM.docx]

**Appendix D** Items included in each domain of PROBAST quality

PROBAST **M. JACOB ET AL. (2020).**

**1. PARTICIPANTS**

**1.1** Were appropriate data sources used, e.g., cohort, randomized controlled trial, or nested case–control study data? **No.**

**1.2** Were all inclusions and exclusions of participants appropriate? **No.**

**Applicability: No.**

**2. PREDICTORS**

**2.1** Were predictors deﬁned and assessed in a similar way for all participants? **Yes.**

**2.2** Were predictor assessments made without knowledge of outcome data? **Unclear**.

**2.3** Are all predictors available at the time the model is intended to be used? **Yes**.

**Applicability**: **Unclear**.

**3. OUTCOME**

**3.1** Was the outcome determined appropriately? **No**.

**3.2** Was a prespeciﬁed or standard outcome deﬁnition used? **Yes.**

**3.3** Were predictors excluded from the outcome deﬁnition? **Yes**.

**3.4** Was the outcome deﬁned and determined in a similar way for all participants? **Yes**.

**3.5** Was the outcome determined without knowledge of predictor information? **Unclear**.

**3.6** Was the time interval between predictor assessment and outcome determination appropriate? **Unclear**.

**Applicability**: **No.**

**4. ANALYSIS**

**4.1** Were there a reasonable number of participants with the outcome? **No** (10.6 EPV).

**4.2** Were continuous and categorical predictors handled appropriately? **No**.

**4.3** Were all enrolled participants included in the analysis? **Yes**.

**4.4** Were participants with missing data handled appropriately? **Unclear**.

**4.5** Was selection of predictors based on univariable analysis avoided? (Model development studies only)? **Yes**.

**4.6** Were complexities in the data (e.g., censoring, competing risks, sampling of control participants) accounted for appropriately? **Yes**.

**4.7** Were relevant model performance measures evaluated appropriately? **No**.

**4.8** Were model overﬁtting and optimism in model performance accounted for? (Model development studies only) **No**.

**4.9** Do predictors and their assigned weights in the ﬁnal model correspond to the results from the reported multivariable analysis? (Model development studies only) **No**.

PROBAST **CHEN W ET AL. (2020).**

**1. PARTICIPANTS**

**1.1** Were appropriate data sources used, e.g., cohort, randomized controlled trial, or nested case–control study data? **No**.

**1.2** Were all inclusions and exclusions of participants appropriate? **No**.

**Applicability: No.**

**2. PREDICTORS**

**2.1** Were predictors deﬁned and assessed in a similar way for all participants? **Yes**.

**2.2** Were predictor assessments made without knowledge of outcome data? **Unclear**.

**2.3** Are all predictors available at the time the model is intended to be used? **Yes**.

**Applicability**: **Unclear**.

**3. OUTCOME**

**3.1** Was the outcome determined appropriately? **No**.

**3.2** Was a prespeciﬁed or standard outcome deﬁnition used? **Yes**.

**3.3** Were predictors excluded from the outcome deﬁnition? **Yes**.

**3.4** Was the outcome deﬁned and determined in a similar way for all participants? **Yes**.

**3.5** Was the outcome determined without knowledge of predictor information? **Unclear**.

**3.6** Was the time interval between predictor assessment and outcome determination appropriate? **Unclear**.

**Applicability**: **No.**

**4. ANALYSIS**

**4.1** Were there a reasonable number of participants with the outcome? **Yes** (40 EPV).

**4.2** Were continuous and categorical predictors handled appropriately? **Yes**.

**4.3** Were all enrolled participants included in the analysis? **Yes**.

**4.4** Were participants with missing data handled appropriately? **Unclear**.

**4.5** Was selection of predictors based on univariable analysis avoided? (Model development studies only)? **Unclear**.

**4.6** Were complexities in the data (e.g., censoring, competing risks, sampling of control participants) accounted for appropriately? **Yes**.

**4.7** Were relevant model performance measures evaluated appropriately? **No**.

**4.8** Were model overﬁtting and optimism in model performance accounted for? (Model development studies only) **No**.

**4.9** Do predictors and their assigned weights in the ﬁnal model correspond to the results from the reported multivariable analysis? (Model development studies only) **No**.

PROBAST **WU Z ET AL. (2020).**

**1. PARTICIPANTS**

**1.1** Were appropriate data sources used, e.g., cohort, randomized controlled trial, or nested case–control study data? **Unclear**.

**1.2** Were all inclusions and exclusions of participants appropriate? **No**.

**Applicability: No.**

**2. PREDICTORS**

**2.1** Were predictors deﬁned and assessed in a similar way for all participants? **Yes**.

**2.2** Were predictor assessments made without knowledge of outcome data? **Unclear**.

**2.3** Are all predictors available at the time the model is intended to be used? **Yes**.

**Applicability**: **Unclear**.

**3. OUTCOME**

**3.1** Was the outcome determined appropriately? **No**.

**3.2** Was a prespeciﬁed or standard outcome deﬁnition used? **Yes**.

**3.3** Were predictors excluded from the outcome deﬁnition? **Yes**.

**3.4** Was the outcome deﬁned and determined in a similar way for all participants? **Yes**.

**3.5** Was the outcome determined without knowledge of predictor information? **Unclear**.

**3.6** Was the time interval between predictor assessment and outcome determination appropriate? **Unclear**.

**Applicability**: **No**.

**4. ANALYSIS**

**4.1** Were there a reasonable number of participants with the outcome? **Unclear**.

**4.2** Were continuous and categorical predictors handled appropriately? **No**.

**4.3** Were all enrolled participants included in the analysis? **No**.

**4.4** Were participants with missing data handled appropriately? **Unclear**.

**4.5** Was selection of predictors based on univariable analysis avoided? (Model development studies only)? **Unclear**.

**4.6** Were complexities in the data (e.g., censoring, competing risks, sampling of control participants) accounted for appropriately? **Yes**.

**4.7** Were relevant model performance measures evaluated appropriately? **Yes**.

**4.8** Were model overﬁtting and optimism in model performance accounted for? (Model development studies only) **Unclear**.

**4.9** Do predictors and their assigned weights in the ﬁnal model correspond to the results from the reported multivariable analysis? (Model development studies only) **Yes**.

PROBAST **CHEN ET AL. (2019).**

**1. PARTICIPANTS**

**1.1** Were appropriate data sources used, e.g., cohort, randomized controlled trial, or nested case–control study data? **No**.

**1.2** Were all inclusions and exclusions of participants appropriate? **No**.

**Applicability**: **No**.

**2. PREDICTORS**

**2.1** Were predictors deﬁned and assessed in a similar way for all participants? **Yes**.

**2.2** Were predictor assessments made without knowledge of outcome data? **Unclear**.

**2.3** Are all predictors available at the time the model is intended to be used? **Yes**.

**Applicability**: **Unclear**.

**3. OUTCOME**

**3.1** Was the outcome determined appropriately? **No**.

**3.2** Was a prespeciﬁed or standard outcome deﬁnition used? **Yes**.

**3.3** Were predictors excluded from the outcome deﬁnition? **Yes**.

**3.4** Was the outcome deﬁned and determined in a similar way for all participants? **Yes**.

**3.5** Was the outcome determined without knowledge of predictor information? **Unclear**.

**3.6** Was the time interval between predictor assessment and outcome determination appropriate? **Unclear**.

**Applicability**: **No**.

**4. ANALYSIS**

**4.1** Were there a reasonable number of participants with the outcome? **No** (88 participants with the outcome).

**4.2** Were continuous and categorical predictors handled appropriately? **No**.

**4.3** Were all enrolled participants included in the analysis? **Yes**.

**4.4** Were participants with missing data handled appropriately? **Yes**.

**4.5** Was selection of predictors based on univariable analysis avoided? (Model development studies only) **Yes**.

**4.6** Were complexities in the data (e.g., censoring, competing risks, sampling of control participants) accounted for appropriately? **Yes**.

**4.7** Were relevant model performance measures evaluated appropriately? **Yes**.

**4.8** Were model overﬁtting and optimism in model performance accounted for? (Model development studies only) **Yes**.

**4.9** Do predictors and their assigned weights in the ﬁnal model correspond to the results from the reported multivariable analysis? (Model development studies only) **Yes**.

PROBAST **WANG ET AL. (2018).**

**1. PARTICIPANTS**

1.1 Were appropriate data sources used, e.g., cohort, randomized controlled trial, or nested case–control study data? **Yes**.

1.2 Were all inclusions and exclusions of participants appropriate? **No**.

**Applicability: No.**

**2. PREDICTORS**

2.1 Were predictors deﬁned and assessed in a similar way for all participants? **Yes**.

2.2 Were predictor assessments made without knowledge of outcome data? **Unclear**.

2.3 Are all predictors available at the time the model is intended to be used? **Yes**.

**Applicability: Unclear.**

**3. OUTCOME**

3.1 Was the outcome determined appropriately? **Yes**.

3.2 Was a prespeciﬁed or standard outcome deﬁnition used? **Yes**.

3.3 Were predictors excluded from the outcome deﬁnition? **Yes**.

3.4 Was the outcome deﬁned and determined in a similar way for all participants? **Yes**.

3.5 Was the outcome determined without knowledge of predictor information? **Unclear**.

3.6 Was the time interval between predictor assessment and outcome determination appropriate? **No**.

**Applicability: No.**

**4. ANALYSIS**

4.1 Were there a reasonable number of participants with the outcome? **Yes** (23.8 EPV).

4.2 Were continuous and categorical predictors handled appropriately? **Yes**.

4.3 Were all enrolled participants included in the analysis? **No**.

4.4 Were participants with missing data handled appropriately? **Unclear**.

4.5 Was selection of predictors based on univariable analysis avoided? (Model development studies only) **Yes**.

4.6 Were complexities in the data (e.g., censoring, competing risks, sampling of control participants) accounted for appropriately? **Yes**.

4.7 Were relevant model performance measures evaluated appropriately? **No**.

4.8 Were model overﬁtting and optimism in model performance accounted for? (Model development studies only) **No**.

4.9 Do predictors and their assigned weights in the ﬁnal model correspond to the results from the reported multivariable analysis? (Model development studies only) **Unclear**.

PROBAST **SHE ET AL. (2017).**

**1. PARTICIPANTS**

**1.1** Were appropriate data sources used, e.g., cohort, randomized controlled trial, or nested case–control study data? **No**.

**1.2** Were all inclusions and exclusions of participants appropriate? **No**.

**Applicability**: **No**.

**2. PREDICTORS**

**2.1** Were predictors deﬁned and assessed in a similar way for all participants? **Yes**.

**2.2** Were predictor assessments made without knowledge of outcome data? **Unclear**.

**2.3** Are all predictors available at the time the model is intended to be used? **Yes**.

**Applicability**: **Unclear**.

**3. OUTCOME**

**3.1** Was the outcome determined appropriately? **Unclear**.

**3.2** Was a prespeciﬁed or standard outcome deﬁnition used? **Yes**.

**3.3** Were predictors excluded from the outcome deﬁnition? **Yes**.

**3.4** Was the outcome deﬁned and determined in a similar way for all participants? **Yes**.

**3.5** Was the outcome determined without knowledge of predictor information? **Unclear**.

**3.6** Was the time interval between predictor assessment and outcome determination appropriate? **Unclear**.

**Applicability**: **No**.

**4. ANALYSIS**

**4.1** Were there a reasonable number of participants with the outcome? **Yes** (86.42 EPV).

**4.2** Were continuous and categorical predictors handled appropriately? **Yes**.

**4.3** Were all enrolled participants included in the analysis? **Yes**.

**4.4** Were participants with missing data handled appropriately? **Unclear**.

**4.5** Was selection of predictors based on univariable analysis avoided? (Model development studies only) **No**.

**4.6** Were complexities in the data (e.g., censoring, competing risks, sampling of control participants) accounted for appropriately? **Yes**.

**4.7** Were relevant model performance measures evaluated appropriately? **Yes**.

**4.8** Were model overﬁtting and optimism in model performance accounted for? (Model development studies only) **Yes**.

**4.9** Do predictors and their assigned weights in the ﬁnal model correspond to the results from the reported multivariable analysis? (Model development studies only) **No**.

PROBAST **YANG ET AL. (2017).**

**1. PARTICIPANTS**

**1.1** Were appropriate data sources used, e.g., cohort, randomized controlled trial, or nested case–control study data? **Unclear**.

**1.2** Were all inclusions and exclusions of participants appropriate? **No**.

**Applicability**: **No**.

**2. PREDICTORS**

**2.1** Were predictors deﬁned and assessed in a similar way for all participants? **Yes**.

**2.2** Were predictor assessments made without knowledge of outcome data? **Unclear**.

**2.3** Are all predictors available at the time the model is intended to be used? **Yes**.

**Applicability**: **Unclear**.

**3. OUTCOME**

**3.1** Was the outcome determined appropriately? **Unclear**.

**3.2** Was a prespeciﬁed or standard outcome deﬁnition used? **Yes**.

**3.3** Were predictors excluded from the outcome deﬁnition? **Yes**.

**3.4** Was the outcome deﬁned and determined in a similar way for all participants? **Yes**.

**3.5** Was the outcome determined without knowledge of predictor information? **Unclear**.

**3.6** Was the time interval between predictor assessment and outcome determination appropriate? **Unclear**.

**Applicability**: **No**.

**4. ANALYSIS**

**4.1** Were there a reasonable number of participants with the outcome? **Yes** (236 participants with the outcome).

**4.2** Were continuous and categorical predictors handled appropriately? **No**.

**4.3** Were all enrolled participants included in the analysis? **Yes**.

**4.4** Were participants with missing data handled appropriately? **Unclear**.

**4.5** Was selection of predictors based on univariable analysis avoided? (Model development studies only) **Unclear**.

**4.6** Were complexities in the data (e.g., censoring, competing risks, sampling of control participants) accounted for appropriately? **Yes**.

**4.7** Were relevant model performance measures evaluated appropriately? **Unclear.**

**4.8** Were model overﬁtting and optimism in model performance accounted for? (Model development studies only) **No**.

**4.9** Do predictors and their assigned weights in the ﬁnal model correspond to the results from the reported multivariable analysis? (Model development studies only) **Unclear**.

PROBAST **VAN GÓMEZ LÓPEZ ET AL. (2015).**

**1. PARTICIPANTS**

1.1 Were appropriate data sources used, e.g., cohort, randomized controlled trial, or nested case–control study data? **No**.

1.2 Were all inclusions and exclusions of participants appropriate? **No**.

**Applicability: No.**

**2. PREDICTORS**

2.1 Were predictors deﬁned and assessed in a similar way for all participants? **Yes**.

2.2 Were predictor assessments made without knowledge of outcome data? **Unclear**.

2.3 Are all predictors available at the time the model is intended to be used **Yes**.

**Applicability: Unclear.**

**3. OUTCOME**

3.1 Was the outcome determined appropriately? **No**.

3.2 Was a prespeciﬁed or standard outcome deﬁnition used? **Yes**.

3.3 Were predictors excluded from the outcome deﬁnition? **Yes**.

3.4 Was the outcome deﬁned and determined in a similar way for all participants? **Yes**.

3.5 Was the outcome determined without knowledge of predictor information? **Unclear**.

3.6 Was the time interval between predictor assessment and outcome determination appropriate? **Unclear**.

**Applicability: No.**

**4. ANALYSIS**

4.1 Were there a reasonable number of participants with the outcome? **Yes** (20 EPV).

4.2 Were continuous and categorical predictors handled appropriately? **Yes**.

4.3 Were all enrolled participants included in the analysis? **Yes**.

4.4 Were participants with missing data handled appropriately? **Unclear**.

4.5 Was selection of predictors based on univariable analysis avoided? (Model development studies only) **Unclear.**

4.6 Were complexities in the data (e.g., censoring, competing risks, sampling of control participants) accounted for appropriately? **Yes**.

4.7 Were relevant model performance measures evaluated appropriately? **No**.

4.8 Were model overﬁtting and optimism in model performance accounted for? (Model development studies only) **No**.

4.9 Do predictors and their assigned weights in the ﬁnal model correspond to the results from the reported multivariable analysis? (Model development studies only) **Yes**.

PROBAST **ZHENG ET AL. (2015).**

**1. PARTICIPANTS**

**1.1** Were appropriate data sources used, e.g., cohort, randomized controlled trial, or nested case–control study data? **Unclear**.

**1.2** Were all inclusions and exclusions of participants appropriate? **No**.

**Applicability**: **No**.

**2. PREDICTORS**

**2.1** Were predictors deﬁned and assessed in a similar way for all participants? **Yes**.

**2.2** Were predictor assessments made without knowledge of outcome data? **Unclear**.

**2.3** Are all predictors available at the time the model is intended to be used? **Yes**.

**Applicability**: **Unclear**.

**3. OUTCOME**

**3.1** Was the outcome determined appropriately? **Unclear**.

**3.2** Was a prespeciﬁed or standard outcome deﬁnition used? **Yes**.

**3.3** Were predictors excluded from the outcome deﬁnition? **Yes**.

**3.4** Was the outcome deﬁned and determined in a similar way for all participants? **Yes**.

**3.5** Was the outcome determined without knowledge of predictor information? **Unclear**.

**3.6** Was the time interval between predictor assessment and outcome determination appropriate? **Unclear**.

**Applicability**: **No**.

**4. ANALYSIS**

**4.1** Were there a reasonable number of participants with the outcome? **Unclear**.

**4.2** Were continuous and categorical predictors handled appropriately? **No**.

**4.3** Were all enrolled participants included in the analysis? **Unclear**.

**4.4** Were participants with missing data handled appropriately? **Unclear**.

**4.5** Was selection of predictors based on univariable analysis avoided? (Model development studies only) **Yes**.

**4.6** Were complexities in the data (e.g., censoring, competing risks, sampling of control participants) accounted for appropriately? **Yes**.

**4.7** Were relevant model performance measures evaluated appropriately? **Unclear**.

**4.8** Were model overﬁtting and optimism in model performance accounted for? (Model development studies only) **No**.

**4.9** Do predictors and their assigned weights in the ﬁnal model correspond to the results from the reported multivariable analysis? (Model development studies only) **No.**

PROBAST **ZHANG ET AL. (2015).**

**1. PARTICIPANTS**

**1.1** Were appropriate data sources used, e.g., cohort, randomized controlled trial, or nested case–control study data? **No**.

**1.2** Were all inclusions and exclusions of participants appropriate? **No**.

**Applicability**: **No**.

**2. PREDICTORS**

**2.1** Were predictors deﬁned and assessed in a similar way for all participants? **Yes**.

**2.2** Were predictor assessments made without knowledge of outcome data? **Unclear**.

**2.3** Are all predictors available at the time the model is intended to be used? **Yes**.

**Applicability**: **Unclear**.

**3. OUTCOME**

**3.1** Was the outcome determined appropriately? **Unclear**.

**3.2** Was a prespeciﬁed or standard outcome deﬁnition used? **Yes**.

**3.3** Were predictors excluded from the outcome deﬁnition? **Yes**.

**3.4** Was the outcome deﬁned and determined in a similar way for all participants? **Yes**.

**3.5** Was the outcome determined without knowledge of predictor information? **Unclear**.

**3.6** Was the time interval between predictor assessment and outcome determination appropriate? **Unclear**.

**Applicability**: **No**.

**4. ANALYSIS**

**4.1** Were there a reasonable number of participants with the outcome? **No** (72 participants with the outcome).

**4.2** Were continuous and categorical predictors handled appropriately? **Yes**.

**4.3** Were all enrolled participants included in the analysis? **Yes**.

**4.4** Were participants with missing data handled appropriately? **Unclear**.

**4.5** Was selection of predictors based on univariable analysis avoided? (Model development studies only) **No**.

**4.6** Were complexities in the data (e.g., censoring, competing risks, sampling of control participants) accounted for appropriately? **Yes**.

**4.7** Were relevant model performance measures evaluated appropriately? **Unclear**.

**4.8** Were model overﬁtting and optimism in model performance accounted for? (Model development studies only) **No**.

**4.9** Do predictors and their assigned weights in the ﬁnal model correspond to the results from the reported multivariable analysis? (Model development studies only) **Yes**.

PROBAST **DONG ET AL. (2013).**

**1. PARTICIPANTS**

**1.1** Were appropriate data sources used, e.g., cohort, randomized controlled trial, or nested case–control study data? **No**.

**1.2** Were all inclusions and exclusions of participants appropriate? **No**.

**Applicability**: **No**.

**2. PREDICTORS**

**2.1** Were predictors deﬁned and assessed in a similar way for all participants? **Yes**.

**2.2** Were predictor assessments made without knowledge of outcome data? **Unclear**.

**2.3** Are all predictors available at the time the model is intended to be used? **Yes**.

**Applicability**: **Unclear**.

**3. OUTCOME**

**3.1** Was the outcome determined appropriately? **No**.

**3.2** Was a prespeciﬁed or standard outcome deﬁnition used? **Yes**.

**3.3** Were predictors excluded from the outcome deﬁnition? **Yes**.

**3.4** Was the outcome deﬁned and determined in a similar way for all participants? **Yes**.

**3.5** Was the outcome determined without knowledge of predictor information? **Unclear**.

**3.6** Was the time interval between predictor assessment and outcome determination appropriate? **Unclear**.

**Applicability**: **No**.

**4. ANALYSIS**

**4.1** Were there a reasonable number of participants with the outcome? **Yes** (117.81 EPV).

**4.2** Were continuous and categorical predictors handled appropriately? **Yes**.

**4.3** Were all enrolled participants included in the analysis? **Unclear**.

**4.4** Were participants with missing data handled appropriately? **Unclear**.

**4.5** Was selection of predictors based on univariable analysis avoided? (Model development studies only) **Yes**.

**4.6** Were complexities in the data (e.g., censoring, competing risks, sampling of control participants) accounted for appropriately? **Yes**.

**4.7** Were relevant model performance measures evaluated appropriately? **Unclear**.

**4.8** Were model overﬁtting and optimism in model performance accounted for? (Model development studies only) **No**.

**4.9** Do predictors and their assigned weights in the ﬁnal model correspond to the results from the reported multivariable analysis? (Model development studies only) **Yes**.

PROBAST **LI ET AL. (2012).**

**1. PARTICIPANTS**

**1.1** Were appropriate data sources used, e.g., cohort, randomized controlled trial, or nested case–control study data? **No**.

**1.2** Were all inclusions and exclusions of participants appropriate? **No**.

**Applicability**: **No**.

**2. PREDICTORS**

**2.1** Were predictors deﬁned and assessed in a similar way for all participants? **Yes**.

**2.2** Were predictor assessments made without knowledge of outcome data? **Unclear**.

**2.3** Are all predictors available at the time the model is intended to be used? **Yes**.

**Applicability**: **Unclear**.

**3. OUTCOME**

**3.1** Was the outcome determined appropriately? **No**.

**3.2** Was a prespeciﬁed or standard outcome deﬁnition used? **Yes**.

**3.3** Were predictors excluded from the outcome deﬁnition? **Yes**.

**3.4** Was the outcome deﬁned and determined in a similar way for all participants? **Yes**.

**3.5** Was the outcome determined without knowledge of predictor information? **Unclear**.

**3.6** Was the time interval between predictor assessment and outcome determination appropriate? **Unclear**.

**Applicability**: **No**.

**4. ANALYSIS**

**4.1** Were there a reasonable number of participants with the outcome? **No** (98 participants with the outcome).

**4.2** Were continuous and categorical predictors handled appropriately? **Yes**.

**4.3** Were all enrolled participants included in the analysis? **Yes**.

**4.4** Were participants with missing data handled appropriately? **Unclear**.

**4.5** Was selection of predictors based on univariable analysis avoided? (Model development studies only) **Unclear**.

**4.6** Were complexities in the data (e.g., censoring, competing risks, sampling of control participants) accounted for appropriately? **Yes**.

**4.7** Were relevant model performance measures evaluated appropriately? **Unclear**.

**4.8** Were model overﬁtting and optimism in model performance accounted for? (Model development studies only) **No**.

**4.9** Do predictors and their assigned weights in the ﬁnal model correspond to the results from the reported multivariable analysis? (Model development studies only) **Yes**.

PROBAST **YONEMORI ET AL. (2007).**

**1. PARTICIPANTS**

**1.1** Were appropriate data sources used, e.g., cohort, randomized controlled trial, or nested case–control study data? **No**.

**1.2** Were all inclusions and exclusions of participants appropriate? **No**.

**Applicability**: **No**.

**2. PREDICTORS**

**2.1** Were predictors deﬁned and assessed in a similar way for all participants? **Yes**.

**2.2** Were predictor assessments made without knowledge of outcome data? **Yes**.

**2.3** Are all predictors available at the time the model is intended to be used? **Yes**.

**Applicability**: **Unclear**.

**3. OUTCOME**

**3.1** Was the outcome determined appropriately? **No**.

**3.2** Was a prespeciﬁed or standard outcome deﬁnition used? **Yes**.

**3.3** Were predictors excluded from the outcome deﬁnition? **Yes**.

**3.4** Was the outcome deﬁned and determined in a similar way for all participants? **Yes**.

**3.5** Was the outcome determined without knowledge of predictor information? **Unclear**.

**3.6** Was the time interval between predictor assessment and outcome determination appropriate? **Unclear**.

**Applicability**: **No**.

**4. ANALYSIS**

**4.1** Were there a reasonable number of participants with the outcome? **Yes** (131.72 EPV).

**4.2** Were continuous and categorical predictors handled appropriately? **Yes**.

**4.3** Were all enrolled participants included in the analysis? **No**.

**4.4** Were participants with missing data handled appropriately? **Unclear**.

**4.5** Was selection of predictors based on univariable analysis avoided? (Model development studies only) **No**.

**4.6** Were complexities in the data (e.g., censoring, competing risks, sampling of control participants) accounted for appropriately? **Yes**.

**4.7** Were relevant model performance measures evaluated appropriately? **Unclear**.

**4.8** Were model overﬁtting and optimism in model performance accounted for? (Model development studies only) **No**.

**4.9** Do predictors and their assigned weights in the ﬁnal model correspond to the results from the reported multivariable analysis? (Model development studies only) **Unclear**.

PROBAST **GOULD ET AL. (2007).**

**1. PARTICIPANTS**

**1.1** Were appropriate data sources used, e.g., cohort, randomized controlled trial, or nested case–control study data? **Yes**.

**1.2** Were all inclusions and exclusions of participants appropriate? **No**.

**Applicability: No.**

**2. PREDICTORS**

**2.1** Were predictors deﬁned and assessed in a similar way for all participants? **No**.

**2.2** Were predictor assessments made without knowledge of outcome data? **Unclear**.

**2.3** Are all predictors available at the time the model is intended to be used? **Yes**.

**Applicability: Unclear.**

**3. OUTCOME**

**3.1** Was the outcome determined appropriately? **Unclear**.

**3.2** Was a prespeciﬁed or standard outcome deﬁnition used? **Yes**.

**3.3** Were predictors excluded from the outcome deﬁnition? **Yes**.

**3.4** Was the outcome deﬁned and determined in a similar way for all participants? **Yes**.

**3.5** Was the outcome determined without knowledge of predictor information? **Unclear**.

**3.6** Was the time interval between predictor assessment and outcome determination appropriate? **No**.

**Applicability**: **No**.

**4. ANALYSIS**

**4.1** Were there a reasonable number of participants with the outcome? **Yes** (51 EPV).

**4.2** Were continuous and categorical predictors handled appropriately? **Unclear**.

**4.3** Were all enrolled participants included in the analysis? **Yes**.

**4.4** Were participants with missing data handled appropriately? **Unclear**.

**4.5** Was selection of predictors based on univariable analysis avoided? (Model development studies only) **Yes**.

**4.6** Were complexities in the data (e.g., censoring, competing risks, sampling of control participants) accounted for appropriately? **Yes**.

**4.7** Were relevant model performance measures evaluated appropriately? **Yes**.

**4.8** Were model overﬁtting and optimism in model performance accounted for? (Model development studies only) **No**.

**4.9** Do predictors and their assigned weights in the ﬁnal model correspond to the results from the reported multivariable analysis? (Model development studies only) **Yes**.

PROBAST **SWENSEN ET AL. (1996).**

**1. PARTICIPANTS**

**1.1** Were appropriate data sources used, e.g., cohort, randomized controlled trial, or nested case–control study data? **Yes**.

**1.2** Were all inclusions and exclusions of participants appropriate? **Yes**.

**Applicability**: **Yes**.

**2. PREDICTORS**

**2.1** Were predictors deﬁned and assessed in a similar way for all participants? **Yes**.

**2.2** Were predictor assessments made without knowledge of outcome data? **Unclear**.

**2.3** Are all predictors available at the time the model is intended to be used? **Yes**.

**Applicability**: **Unclear**.

**3. OUTCOME**

**3.1** Was the outcome determined appropriately? **Yes**.

**3.2** Was a prespeciﬁed or standard outcome deﬁnition used? **Yes**.

**3.3** Were predictors excluded from the outcome deﬁnition? **Yes**.

**3.4** Was the outcome deﬁned and determined in a similar way for all participants? **Yes**.

**3.5** Was the outcome determined without knowledge of predictor information? **Unclear**.

**3.6** Was the time interval between predictor assessment and outcome determination appropriate? **No**.

**Applicability**: **No**.

**4. ANALYSIS**

**4.1** Were there a reasonable number of participants with the outcome? **Unclear**.

**4.2** Were continuous and categorical predictors handled appropriately? **Yes.**

**4.3** Were all enrolled participants included in the analysis? **Yes**.

**4.4** Were participants with missing data handled appropriately? **Unclear**.

**4.5** Was selection of predictors based on univariable analysis avoided? (Model development studies only) **Yes**.

**4.6** Were complexities in the data (e.g., censoring, competing risks, sampling of control participants) accounted for appropriately? **Yes**.

**4.7** Were relevant model performance measures evaluated appropriately? **Yes**.

**4.8** Were model overﬁtting and optimism in model performance accounted for? (Model development studies only) **No**.

**4.9** Do predictors and their assigned weights in the ﬁnal model correspond to the results from the reported multivariable analysis? (Model development studies only) **Yes**.

According to PROBAST, the answers to each item of the domains should be answered as follows: "Probably yes", "Probably no", "Yes", "No", "No information" or "Unclear". We, by consensus, have answered the questions avoiding the first two answers, given the difficult definition of them objectively, therefore, the categories have been "Yes", "No" and "Unclear".
